# Mandibular Overdenture Supported by Two or Four Unsplinted or Two Splinted Ti-Zr Mini-Implants: In Vitro Study of Peri-Implant and Edentulous Area Strains

**DOI:** 10.3390/biomimetics9030178

**Published:** 2024-03-15

**Authors:** Dario Puljic, Nikola Petricevic, Asja Celebic, Ines Kovacic, Manuela Milos, Dalibor Pavic, Ognjen Milat

**Affiliations:** 1 Department of Removable Prosthodontics, University of Zagreb School of Dental Medicine, 10000 Zagreb, Croatia; petricevic@sfzg.hr (N.P.); kovacic@sfzg.hr (I.K.); mmilos@sfzg.hr (M.M.); dpavic@sfzg.hr (D.P.); 2 Institute of Physics, 10000 Zagreb, Croatia; ognjen@milat.hr

**Keywords:** Ti-Zr mini dental implants, strain gauges, mandibular overdenture, loading forces, two MDIs, splinting status

## Abstract

Clinical indications for the newly released Ti-Zr (Roxolid^®^) alloy mini-implants (MDIs) aimed for overdenture (OD) retention in subjects with narrow alveolar ridges are not fully defined. The aim of this study was to analyze peri-implant and posterior edentulous area microstrains utilizing models of the mandible mimicking a “real” mouth situation with two (splinted with a bar or as single units) or four unsplinted Ti-Zr MDIs. The models were virtually designed from a cone beam computed tomography (CBCT) scan of a convenient patient and printed. The artificial mucosa was two millimeters thick. After MDI insertion, the strain gauges were bonded on the oral and vestibular peri-implant sites, and on distal edentulous areas under a denture. After attaching the ODs to MDIs, the ODs were loaded using a metal plate positioned on the first artificial molars (posterior loadings) bilaterally and unilaterally with 50, 100, and 150 N forces, respectively. During anterior loadings, the plate was positioned on the denture’s incisors and loaded with 50 and 100 N forces. Each loading was repeated 15 times. The means with standard deviations, and the significance of the differences (two- and three-factor MANOVA) were calculated. Variations in the MDI number, location, and splinting status elicited different microstrains. Higher loading forces elicited higher microstrains. Unilateral loadings elicited higher microstrains than bilateral and anterior loadings, especially on the loading side. Peri-implant microstrains were lower in the four-MDI single-unit model than in both two-MDI models (unsplinted and splinted). Posterior implants showed higher peri-implant microstrains than anterior in the four-MDI model. The splinting of the two-MDI did not have a significant effect on peri-implant microstrains but elicited lower microstrains in the posterior edentulous area. The strains did not exceed the bone reparatory mechanisms, although precaution and additional study should be addressed when two Ti-Zr MDIs support mandibular ODs.

## 1. Introduction

Mini dental implants (MDIs), listed in Category 1 of narrow implants with a diameter of ≤2.5 mm [1], manufactured from Ti90Al6V4 alloy have a Grade 5 tensile strength, which is higher than that of commercially pure Ti (Grade 4). The strength of Grade 4 pure titanium is insufficient for small-diameter dental implants [2]. Higher grades of titanium alloys reduce the risk of implant fracture, especially in narrow implants [3].

Over the last three decades, the placement of four MDIs (Grade 5, Ti90Al6V4 alloy) in the intraforaminal region has proven to be a successful treatment option for the retention and support of mandibular overdentures (ODs). Most clinical studies reported survival rates ranging from 95 to 100%, small amounts of peri-implant bone loss, and increased patients’ satisfaction [4,5,6,7,8,9,10,11,12,13,14,15,16]. A recent study found out that even only three MDIs can be sufficient for satisfactory five-year clinical outcomes [17].

One study, however, with a limited sample size, reported increased seven-year satisfaction in patients rehabilitated with only two MDIs retaining a mandibular OD [18]. Another study, including only eleven patients, reported the one-year increased masticatory efficiency with only two MDIs supporting mandibular ODs [19]. Jofre et al. [20] reported higher amounts of peri-implant bone loss in patients with two unsplinted MDIs supporting a mandibular OD after 15 months in function (1.40 ± 1.02 mm), than in patients with two splinted MDIs (0.84 ± 0.66 mm). Therefore, they recommended the splinting of two mini-implants, or the insertion of four MDIs as single units. However, the duration of that study was limited. The insertion of only two mini-implants showed sufficient success rates over five years only when used for partial removable denture retention [21,22].

Today, the dental market, in addition to the TiAl6V4 alloy MDIs, also offers the recently released Roxolid^®^ alloy (15% zirconium and 85% titanium) MDIs patented by the Straumann Group (Straumann^®^ Mini Implant System). The Ti-Zr mini-implants are available in only one diameter (2.4 mm). However, their length can be either 10 mm, 12 mm, or 14 mm. The Roxolid^®^ alloy has a high tensile strength and excellent osseointegration capabilities [23,24,25]. The new mini-implants are apically tapered, similar to the mini-implants made of TiAl6V4 alloy. This tapered form allows for the underpreparation of the bone and the achievement of a high primary stability during implant insertion. The new Roxolid^®^ mini-implant system has some modifications and innovations compared to TiAl6V4 alloy MDIs. Instead of the metal housing with a resilient rubber “O” ring for OD retention, the new system has an Optiloc^®^ prosthetic connection. A carbon-based prosthetic connection coating covers the surface of the balled-heads of Ti-Zr MDIs (amorphous diamond-like carbon, or ADLC), enabling excellent wear resistance when attached to a female PEEK matrix insert, incorporated in a titanium housing [26]. Another advantage of the Roxolid^®^ mini-implant system is the variability in the dimensions of the polished transmucosal part, allowing the choice of a transmucosal height dependent on the dimensions of the attached mucosa. Other mini-implant systems usually have only one dimension of the transmucosal part.

The novel Roxolid^®^ tissue-level mini-implant system has been on the dental market since 2020; therefore, only a few publications on it are available in the dental literature [27,28]. The manufacturer recommends the insertion of four Roxolid^®^ MDIs for mandibular OD retention and support. The one-year study reported that the success rate of four single-unit Ti-Zr MDIs loaded by a mandibular OD was 100% in 74 patients, presenting a predictable treatment option even when flapless surgery and immediate loading were adopted [28]. However, the lack of studies dealing with the new Ti-Zr MDIs and the short period since the new system appeared on the market indicate the need for research at the in vitro level to obtain new knowledge for clinical application, especially when the insertion of less than four MDIs is being considered as an option. In vitro studies offer important insights into the biomechanics of the new Ti-Zr MDIs, since long-term clinical studies are lacking. The improved design of the Ti-Zr MDIs made of alpha alloy (close-packed hexagonal crystals) and biomechanical properties, in contrast to the Ti90Al6V4 alloy consisting of an alpha and beta phase (close-packed hexagonal and body-centered cubic crystals) [29], could reduce microstrains around the implants’ neck caused by OD loading and improve the stability of the peri-implant bone.

Moreover, the results could contribute to the further progress of clinical practice and knowledge about the possibility of using only two mini-implants for OD retention as a less invasive treatment option in implantology. The hypothesis was that, by increasing the loading force, the amount of microstrains will, consequently, increase, not only around the implants, but also in the posterior edentulous areas under the denture saddles. Another hypothesis was that the splinting of two MDIs would reduce peri-implant and edentulous area microstrains. Because the new Ti-Zr MDIs offer superior mechanical strength, excellent osseointegration, and the reduced risk of fracture [30], we designed this study to find out whether only two Ti-Zr MDIs, either as single units or splinted, can successfully be used instead of four single-unit MDIs. By inserting a smaller number of implants, mini-implant-retained ODs would be financially more acceptable, allowing a larger number of patients to be able to afford it. For that purpose, this in vitro study was designed, trying to mimic, as much as possible, the real situation. The aim was to register and compare peri-implant microstrains in two splinted, two unsplinted, and four unsplinted Ti-Zr MDIs retaining a mandibular OD, under different loads applied at different OD positions. Another goal was to analyze the strains in the posterior edentulous OD-bearing area under denture saddles.

## 2. Materials and Methods

### 2.1. Models of an Atrophied Mandible with a Narrow Alveolar Ridge

The models of the completely edentulous atrophic mandible with a narrow alveolar ridge were fabricated based on the CBCT (ProMax 3D, Planmeca, Helsinki, Finland) scans of a convenient patient with the atrophied edentulous mandible after the written approval obtained from the patient and with the approval of the Institution Ethics Committee (No. 05-PA-26-6/2015). Based on the CBCT scans, a virtual model was created in the Amira software (Amira, v4.1, Zuse Institute Berlin; Visage Imaging GmbH, Berlin, Germany). The Blender^®^ software (Blender^®^, v2.79b, Amsterdam, The Netherlands) was used to plan the positions of the mini-dental implants in the interforaminal area in order to design holes for their insertion. According to the number of MDIs planned for insertion, two types of models were designed, one model with four holes for the insertion of four MDIs (in positions of the previous teeth 44, 42, 32, and 34) and another two models with two holes for the insertion of two MDIs (in the positions of the previous teeth 33 and 43). The length of the holes for the MDI insertion was 10 mm (the entire length of the MDI-roughened surface with threads), but the width was 2.3 mm, which is 0.1 mm narrower than the diameter of the implant body at the bone interface. The narrower diameter of the holes aimed to ensure the stability of the inserted MDIs.

Stereolithographic 3D-printing technology (Form 2, Formlabs, Somerville, MA, USA) and Gray photopolymer resin (GRAY FLGPGR04; Formlabs, Somerville, MA, USA) were used to create three models of the same mandible, one with four holes, and two with two holes. After printing, further processing included immersion of the models in 95% isopropyl alcohol (IPA) (Izopropil alkohol, Medimon d.o.o., Split, Croatia) for one minute, and then additionally for 15 min in a new container of IPA to rinse the residual resin. After cleaning, 30 min polymerization with 36 W UV-A halogen lights (Dentsply Sirona Heliodent Plus, Display Sirona, York, PA, USA, SAD) and 30 min heating in a chamber at 60 °C were performed for each model. All models had mechanical properties similar to D2 bone.

### 2.2. Artificial Mucosa Fabrication, Implant Insertion, and Overdenture Fabrication

An artificial mucosa of equal thickness (2 mm) was made for each mandibular model from the vinyl polysiloxane impression material (3M™ Express™ XT Light Body Quick, Seefeld, Germany). To ensure the uniform thickness, molds for injecting the impression material were designed. The molds were designed virtually (Amira v4.1, Zuse Institute Berlin; Visage Imaging GmbH, Berlin, Germany) for the 2.0 mm-thick mucosa, but with different number of perforations. The molds had either two or four perforations at the sites where the insertion of a mini-implant was planned. The impression material (a-silicone) was mixed and injected into each mold under manual pressure, and, after material setting, the artificial mucosa was taken out and placed over the model of the mandible.

In each of the three models, two or four MDIs with a diameter of 2.4 mm and a length of 10 mm (Straumann^®^ Mini Implant, Institute Straumann AG, Basel, Switzerland) were inserted. The gingival height of 2.8 mm (i.e., the height of polished neck of the implant) was chosen among the Straumann^®^ Mini Implants for the artificial mucosa height of 2 mm.

The MDIs were inserted, first, using a vial cap as a finger driver, and, afterwards, using a manual ratchet to finish screwing it into the hole until the whole roughened threaded implant surface was inside the model. Insertion torque on the ratchet (BLX Torque Control Device, Institute Straumann AG, Basel, Switzerland) varied for a very small amount (5 Ncm) between different insertion sites. The manufacturer recommends torque values of ≥35 Ncm for immediate loading. All torque values obtained during insertion of MDIs in this study were higher than 35 Ncm.

**Figure 1:**
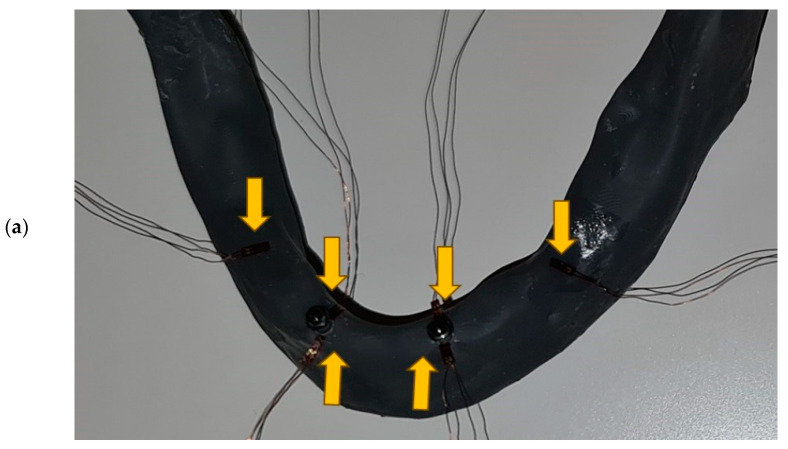

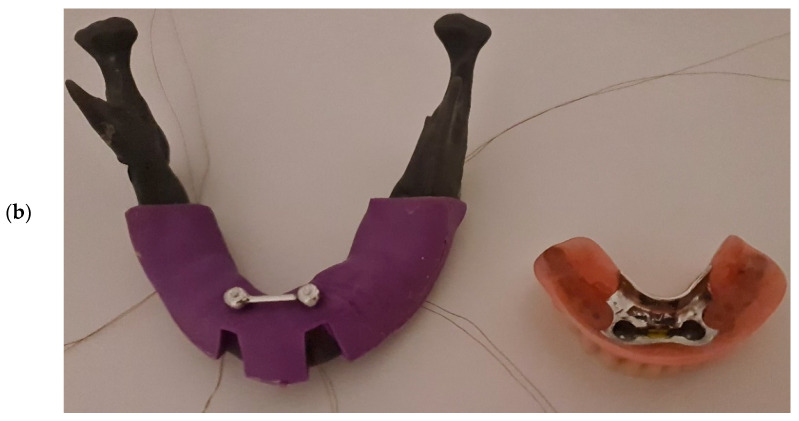
(**a**) Strain gauges bonded on the vestibular and oral side of “bone”, as close as possible to the mini-implants, and on the distal posterior edentulous area (under overdenture saddle) in the two-MDI unsplinted model (Yellow arrows show SGs); (**b**) the 2 splinted MDIs with strain gauges, a silicone mask mimicking 2 mm-thick mucosa (left side), and the respective overdenture with a yellow ready-made clip (right side).

After insertion of all MDIs, the models with artificial mucosa were scanned with the laboratory scanner (3Shape 3E, 3Shape, Copenhagen, Denmark) for overdenture manufacturing. The four-MDI models had four single-unit MDIs. One model was left with two MDIs as single units (not splinted), while, in another model, two MDIs were splinted with a bar (Figure 1b, left side), which was also designed in the same 3Shape software. After modeling a bar using computer-aided design (CAD) in the 3Shape software (3Shape, v20.1, Copenhagen, Denmark), the bar was milled from BEGO Mediloy^®^ M-Co (BEGO, Bremen, Germany) with the Imes-icore CORiTEC 350i machine (GmbH, Eiterfeld, Germany). The bar was cemented on the implant necks using the self-curing and self-etching adhesive cement (Maxcem Elite^TM^ Self-Etch/Self Adhesive Resin Cement, KaVo Kerr, Brea, CA, USA) (Figure 1b).

A design of the metal framework which was manufactured to strengthen a denture was carried out using computer-aided design (CAD) technology in the 3Shape software (3Shape, v.20.1, Copenhagen, Denmark). Three metal frameworks were created, one for the unsplinted model with four MDIs, one for the model with two splinted MDIs (Figure 1b, right side), and one for the model with two single-unit (unsplinted) MDIs. Metal frameworks were manufactured from Wironium^®^ RP metal powder (BEGO, Bremen, Germany) using the Sisma Mysint100 Dual Laser (Sisma, Piovene Rocchette, Italy) (additive manufacturing, laser sintering).

Further overdenture fabrication followed the well-known procedure of teeth setting (Ivostar, Ivoclar Vivadent, Schaan, Liechtenstein) in wax rims in a dental laboratory, and final processing of the acrylic resin according to the manufacturer’s recommendation (Ivoclar ProBase Hot Denture Resin, Ivoclar Vivadent, Schaan, Liechtenstein) and polishing of the overdenture. The metal housings of the Optiloc^®^ (Institute Straumann AG, Basel, Switzerland) retention matrices with the medium (yellow) PEEK retention inserts (1200 g of the retention force, each) were built in the overdenture simultaneously with the overdenture polymerization in the two- and four-MDI single-unit (not splinted) MDI models. In the two-MDI splinted model, ready-made yellow plastic clips were used (CEKA, PRECI-HORIX COMBI, Waregem, Belgium) for retention of the respective OD (Figure 1b right side), allowing a rotational and a slight vertical movement of the attached OD by compression of the plastic riders.

### 2.3. Strain Gauges Placement, Model Fixation, Overdenture Loading, and Microstrain Measurement

Strain gauges (SG) (KFGS-1N-120-C1-11N30C2, Kyowa Electronic Instruments Co., Ltd., Tokyo, Japan) were used to measure microstrains on the surface of the peri-implant bone and on distal edentulous jaw under free-end saddles (Figure 1a). According to the manufacturer’s instructions, cyanoacrylate adhesive (Super Glue, NU Co., Ltd., Ningbo, China) and acetate foil (Grafix Clear Acetate, Grafix^®^ Plastics, Pennsylvania, USA) were used to bond the strain gauges. Before bonding, the surface of the 3D-printed mandible was cleaned with acetone (Aceton, Premifab d.o.o., Sveta Nedelja, Croatia) to allow better adhesion. Strain gauges were placed near the neck of each MDI, one SG on the vestibular and another SG on the oral side of each MDI (Figure 1a). Another pair of SGs was placed distally, on both sides of the edentulous mandible in the previous sites of the second molars, under the posterior end of the free-end overdenture saddles, to register strains from the posterior edentulous OD-bearing area when the OD was loaded (Figure 1a,b).

Through the compact recording system (EDX-10A v02.00, Kyowa Electronic Instruments Co., Ltd., Tokyo, Japan), all strain gauges were connected to the corresponding software program (DCS-100A v4.6, Kyowa Electronic Instruments Co., Ltd., Tokyo, Japan) that allowed simultaneous monitoring and recording of deformations during measurement.

**Figure 2:**
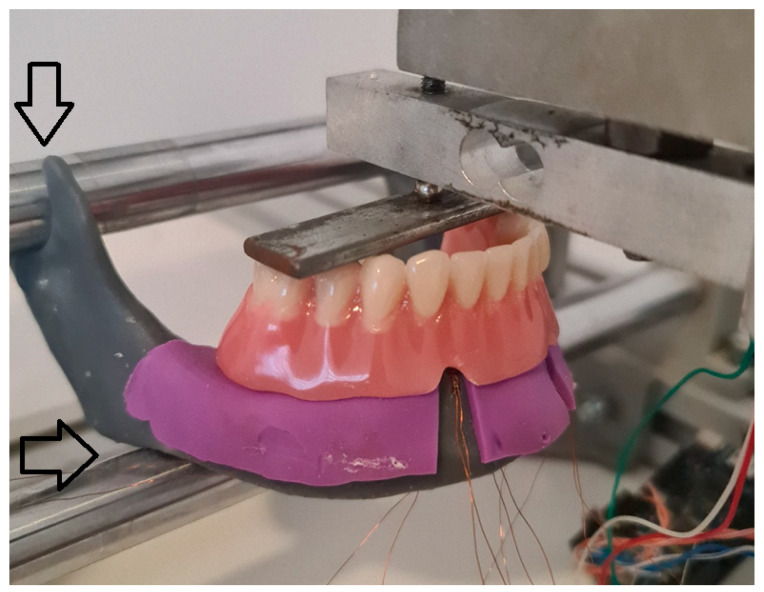
Mandibular 2-MDI model mounted in the stand to mimic the position of the mandible related to skull. The overdenture was loaded bilaterally through the metal plate placed on the denture molar teeth. Arrows point out areas where the model was attached to the stand.

The special stand was constructed for fixation of the models of the mandible simulating its position related to skull. The stand was made of aluminum profile framework: two round bars were placed horizontally within the framework to support the model on the areas corresponding to the insertion of the masseter muscles (Figure 2, shown with a horizontal arrow), and in the mandibular notch (the concavity between the processus condylaris and processus coronoideus) simulating the temporomandibular joint (Figure 2, pointed with a vertical arrow).

### 2.4. Overdenture Loading

The OD was positioned in the stand and the metal screw was twisted to apply a pressure on the metal plate positioned on the OD’s artificial teeth. The ODs were loaded bilaterally (Figure 2) (the second premolars and the first molars), while the metal screw was connected, at the same time, to a force measuring cell (bilateral loading). In this way, the extent of the applied forces was measured and the uniformity of applied force was ensured. The applied force was measured in the same way during other loadings. Except for the bilateral loading, the ODs were also loaded on the second premolar and the first molar teeth unilaterally, only on the right side of the denture (unilateral loading), and on the OD’s incisors (frontal loading). Briefly, each overdenture was loaded in three different positions: frontally (incisor teeth), bilaterally (second premolars and first molars on both sides of the mandible), and unilaterally (right side premolar and molar). The loadings were performed with three different forces, 50 N, 100 N, and 150 N, respectively, for bilateral and unilateral loading, and with two forces, 50 and 100 N, for frontal loading. Microstrains were registered from peri-implant sites and from distal edentulous area under OD saddles. Peri-implant microstrains were registered from SGs in the proximity of the MDIs (SGs were positioned on the vestibular and oral side as close as possible to each implant). Microstrains from distal edentulous area were registered from the SGs positioned in the posterior edentulous area under free-end OD saddles. Loadings of the ODs were performed during an interval of few seconds until the desired loading force was achieved, and then it was maintained for 2 s. All measurements were repeated fifteen times. Maximum microstrain values recorded from the 2 s interval of each measurement were used in the statistical analysis.

### 2.5. Statistical Analysis

Statistical analysis was carried out using the SPSS 20 software. The one-sample Kolmogorov–Smirnov test was used to test the normality of the distribution. Descriptive statistics (mean values and standard deviations) were calculated. Measured microstrains are also presented in the boxplot diagrams. Significance of the differences of recorded microstrains from the 4-MDI model dependent on the loading position and extent of applied forces was tested by the 2-factor MANOVA and Sheffe post hoc tests. Significance of the differences for recorded microstrains dependent on the loading position, extent of applied forces, and different splinting status in the models with two MDIs (splinted and unsplinted) was tested by the 3-factor MANOVA and Sheffe post hoc tests. Effect sizes were assessed by partial eta-squared (for a comparison of more than two groups) and interpreted as follows: η^2^ = 0.01 indicates a small effect, η^2^ from 0.06 to <0.14 indicates a medium effect, while η^2^ = 0.14 indicates a large effect.

## 3. Results

**Figure 3:**
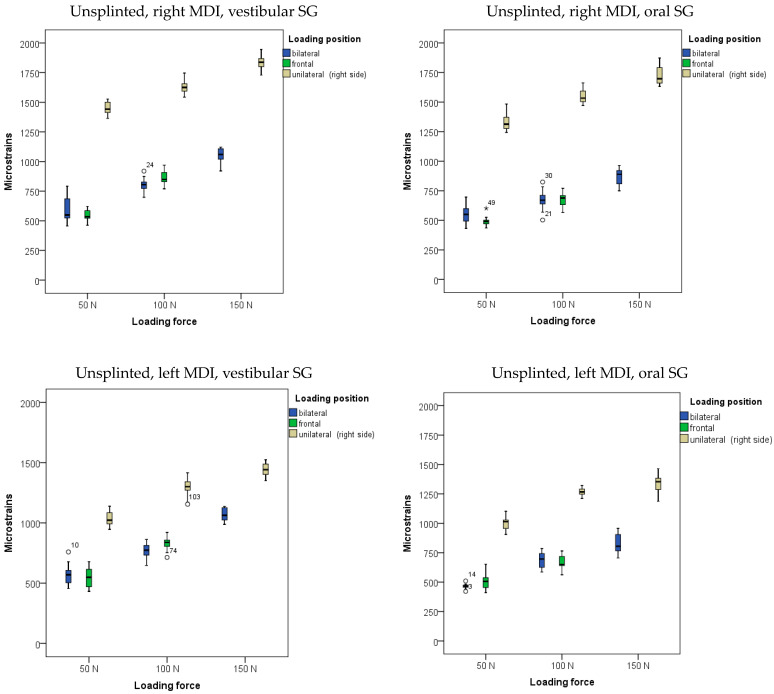
Peri-implant microstrains obtained from two single-unit (unsplinted) mini-implants supporting mandibular overdenture under frontal, bilateral, and unilateral loads of 50, 100, and 150 N, and frontal loads of 50 and 100 N (Open circles represent outliers, asterisks represent extreme outliers).

The descriptive statistics (arithmetic means and standard deviations) of microstrains registered from oral and vestibular peri-implant SGs in the two-MDI single-unit (unsplinted) model during mandibular OD loading are presented in Figure 3 and in Supplementary Table S1.

The highest amounts of microstrains in the two-MDI unsplinted model was obtained from the right peri-implant SG when the OD was unilaterally (on the right side) loaded with a force of 150 N, followed by microstrains from the left peri-implant SG, which were slightly lower. Lower microstrains were also recorded at lower forces and bilateral loads, while the lowest microstrains were recorded at a loading force of 50 N.

**Figure 4:**
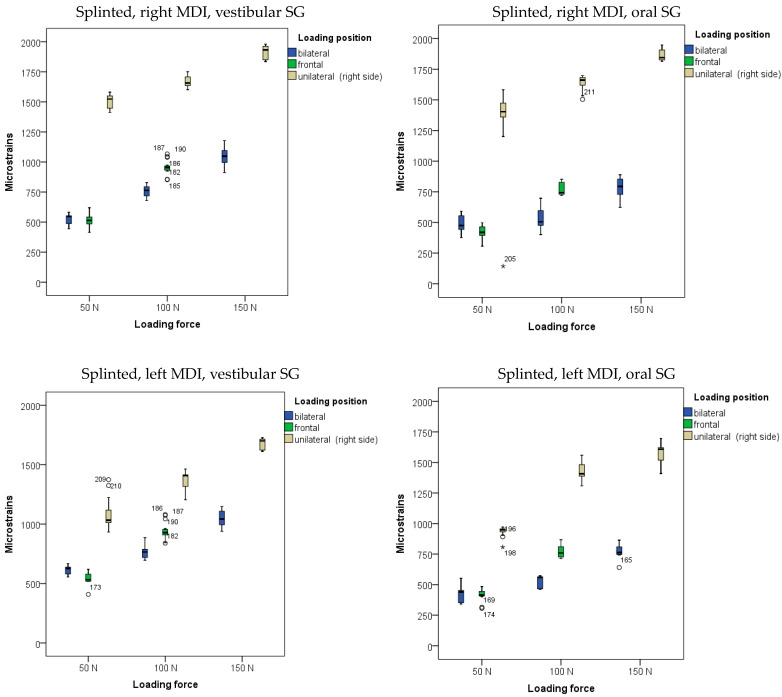
Peri-implant microstrains obtained from two splinted mini-implants supporting mandibular overdenture under frontal, bilateral, and unilateral loads of 50, 100, and 150 N, and frontal loads of 50 and 100 N (Open circles represent outliers, asterisks represent extreme outliers).

The descriptive statistics (arithmetic means and standard deviations) of microstrains obtained from oral and vestibular peri-implant SGs in the two-MDI splinted model during mandibular OD loading are shown in Figure 4, and also in Supplementary Table S1.

The highest microstrains in the two-MDI splinted model were registered from the right peri-implant SGs when the OD was loaded unilaterally (on the right side) with a force of 150 N, followed by the microstrains registered from the left peri-implant SGs (same loading position—right side: unilateral). Lower amounts of microstrains were recorded at lower forces and bilateral loading, while the lowest microstrains were recorded at a force of 50 N and frontal loading. The results of the MANOVA analysis with peri-implant microstrains recorded from the left and right peri-implant vestibular and oral SGs as dependent variables, with loading position, loading force, and splinting status as factors (the three-factor MANOVA), together with the effect size estimation (partial eta-Squared: η^2^), are shown in Supplementary Table S2a. The analysis revealed that the model was significant (*p* < 0.001). The loading forces showed a significant effect on peri-implant microstrain values (*p* < 0.001; higher forces led to higher microstrain values). The effect sizes were large (right side, vestibular SG: η^2^ = 0.902; right side, oral SG: η^2^ = 0.670; left side vestibular SG: η^2^ = 0.886; left side, oral SG: η^2^ = 0.894) (Supplementary Table S2a). The post hoc tests for loading forces are shown in Supplementary Table S2b. All forces were significantly different from each other. The loading position also showed significant effects (*p* < 0.01), as unilateral loading led to the highest microstrains, followed by bilateral and frontal loadings (Supplementary Table S2a). The effect sizes were large (right side, vestibular SG: η^2^ = 0. 979; right side, oral SG: η^2^ = 0.945; left side vestibular SG: η^2^ = 0.927; left side, oral SG: η^2^ = 0.954) (Supplementary Table S2a). The post hoc tests for the loading position are shown in Supplementary Table S2c. However, the splinting status generally did not show significant effects on peri-implant microstrain values (*p* > 0.05; Supplementary Table S2a). Pairwise microstrain comparisons dependent on the splinting status are shown in Supplementary Table S2d.

**Figure 5:**
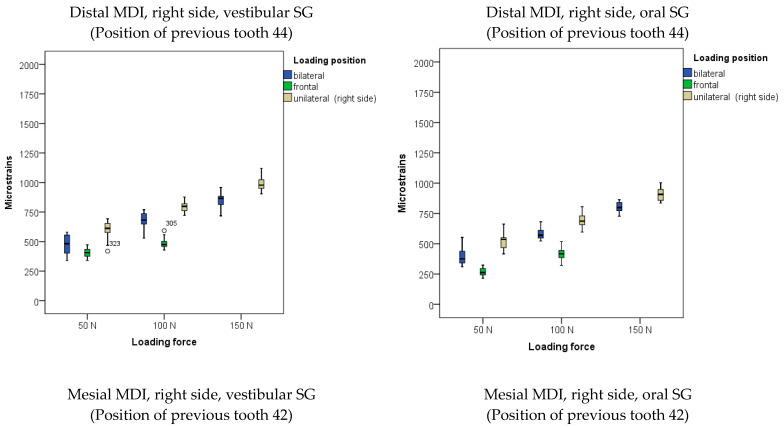

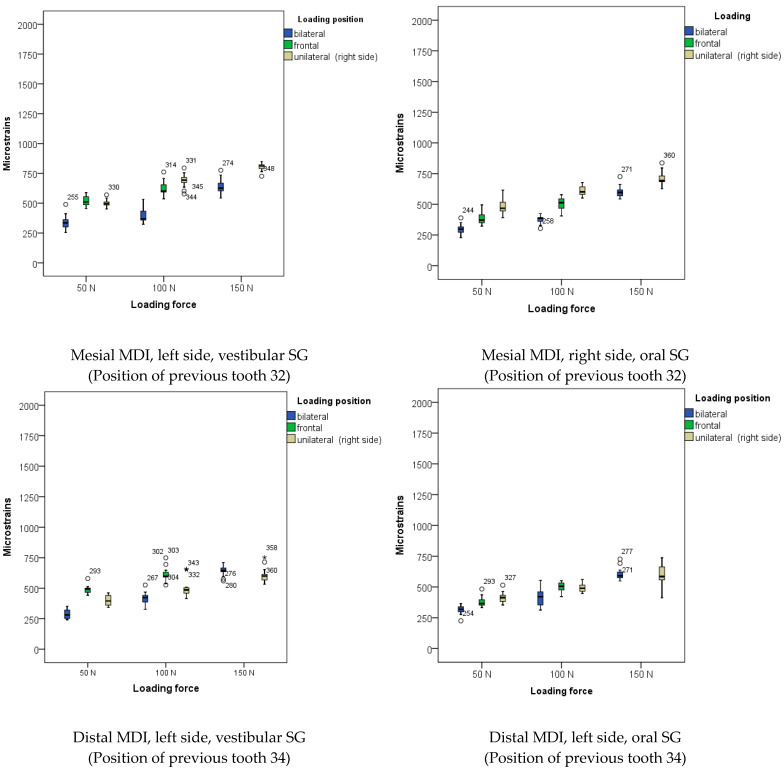

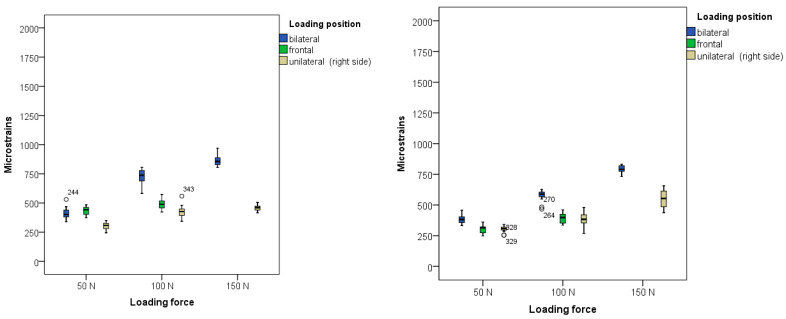
Peri-implant microstrains obtained from four single-unit mini-implants supporting mandibular overdenture under frontal, bilateral, and unilateral loads of 50, 100, and 150 N, and frontal loads of 50 and 100 N (Open circles represent outliers, asterisks represent extreme outliers).

The descriptive statistics (arithmetic means and standard deviations) of microstrains registered from oral and vestibular peri-implant SGs in the four-MDI single-unit (unsplinted) model during mandibular OD loading are presented in Figure 5 and in Supplementary Table S3.

Peri-implant microstrains registered in the four-MDI model (unsplinted) from the vestibular and oral SGs showed lower values than from the two-MDI model (no matter of the splinting status). The maximum strains recorded from the right posterior MDI under 150 N force and unilateral (right-side) loading did not exceed 1000 εμ. The posterior MDIs showed higher values than mesial MDIs.

In the four-MDI unsplinted model, the two-factor MANOVA with peri-implant microstrains (vestibular and oral SGs) registered from the distal and mesial MDIs on the right and left sides of the model as dependent variables with loading forces and loading positions as factors (Supplementary Table S4a–c) showed that the model was significant (*p* < 0.001). All microstrain values obtained were different under different loading forces, with higher forces eliciting higher strains (*p* < 0.001). The effect size was large (partial eta-squared values (η^2^) ranged from 0.762 to 0.976 when the respective OD was loaded with different forces). Supplementary Table S4b shows the post hoc Sheffe tests with the significance of the differences for the factor of loading force. The loading position also elicited significant effects (*p* < 0.001), with the highest strains recorded from the distal right MDI under a 150 N force during unilateral loading. Similar strains were obtained during bilateral loading with a force of 150 N from the left distal MDI. The effect size was large (partial eta-squared values (η^2^) ranged from 0.244 to 0.870 when the OD was loaded at different positions (Supplementary Table S4a)). The post hoc Sheffe tests with the significance of the differences dependent on the loading position are shown in Supplementary Table S4c.

**Figure 6:**
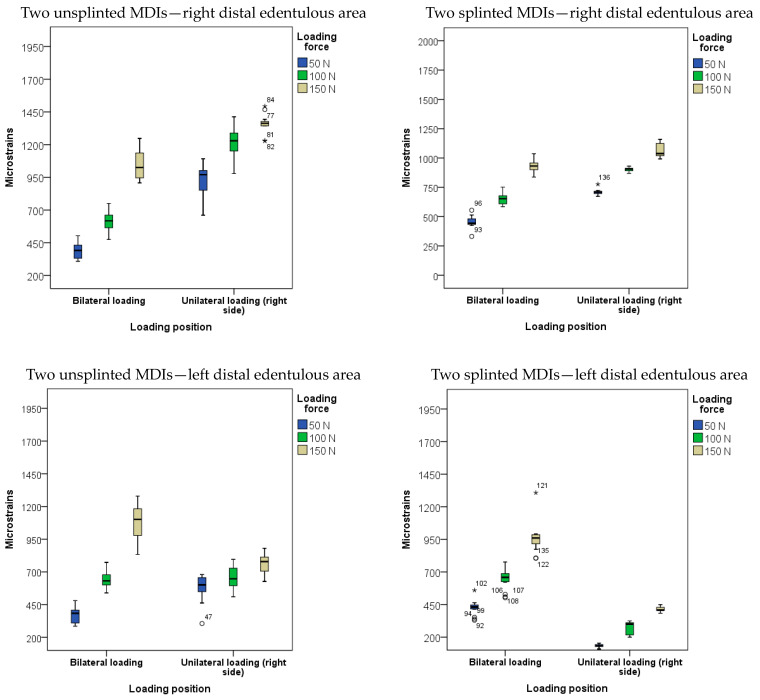
Microstrains obtained from strain gauges in posterior edentulous area under denture saddles during overdenture loading by bilateral and unilateral loadings with forces of 50, 100, and 150 N in the two-MDI unsplinted and splinted models (Open circles represent outliers, Asterisks represent extreme outliers).

The descriptive statistics of microstrains registered from the posterior right and left sides of the edentulous area under the OD saddles in the two-MDI unsplinted, as well as in the two-MDI splinted, models are presented in Figure 6 and in Supplementary Table S5.

The three-factor (loading position, loading force, and splinting status) MANOVA model with the dependent variables of microstrains registered from SGs bonded on the posterior right and left edentulous area, and with the factors of loading force, loading position, and splinting status showed significant effects (*p* < 0.001, Supplementary Table S6a). Higher loading forces elicited higher strains in both edentulous areas during denture settling under loads (*p* < 0.01) with large effect sizes (η^2^): 0.508 on the left side, and 0.868 on the right side, respectively; Supplementary Table S6a). The highest strains were recorded during bilateral and unilateral OD loadings with 150 N forces from the right edentulous area, and under the same force during bilateral loading from the left edentulous area (*p* < 0.01). The effect sizes for the loading position were also large (η^2^): left side = 0.36, right side = 0.835. The splinting status also elicited significant effects, as denture settling mostly elicited higher microstrains in the unsplinted MDI than in the splinted model (*p* < 0.01) with large effect sizes (η^2^ = 0.336 on the left side, and 0.445 on the right side (Supplementary Table S6a)). The post hoc tests for the loading position with the significance of the differences between the mean microstrain values are shown in Supplementary Table S6b. Table S6c shows the significance of the differences dependent on the splinting status (with Sidak adjustments for multiple comparisons). The post hoc tests for the loading force are shown in Supplementary Table S6d.

**Figure 7:**
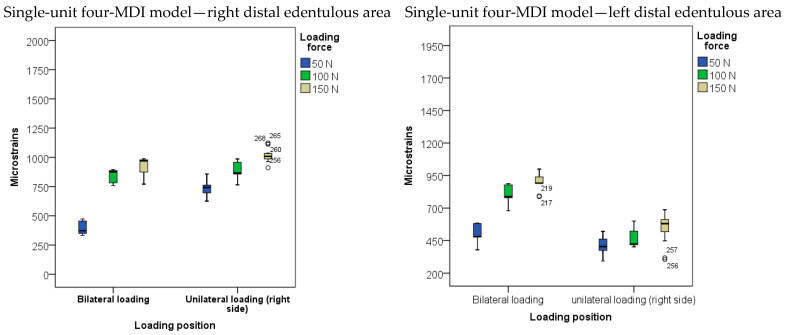
Microstrains obtained from strain gauges in posterior edentulous area under denture saddles during overdenture loading by bilateral and unilateral loadings with forces of 50, 100, and 150 N in the four-MDI model (Open circles represent outliers).

The descriptive statistics of microstrains registered from SGs positioned in the posterior right and left sides of the edentulous area under OD saddles in the four-MDI single-unit mandibular model is presented in Figure 7 and Supplementary Table S7a. The two-factor (loading force and loading position) MANOVA model with the dependent variables strains registered from the posterior right and left edentulous area was significant (*p* < 0.001, Supplementary Table S7b). Significant effects (*p* < 0.01) were elicited by both the loading force and loading position with high effect sizes for both factors (η^2^ = 0.884 and 0.703 for strains registered from the right and the left edentulous sides (the factor of loading force); and 0.639 and 0.753 for the loading position, respectively). The highest strains registered from the distal edentulous area in the four-MDI unsplinted model were similar to those registered in the two-MDI splinted mandibular model. The post hoc tests for microstrains registered from posterior edentulous areas dependent on the loading position are shown in Supplementary Table S7c. The post hoc tests for microstrains registered from posterior edentulous areas dependent on the extent of the loading forces are shown in Supplementary Table S7d.

## 4. Discussion

The use of dental implants in oral rehabilitation has increased, leading to the development of new biomaterials, improvements of existing biomaterials, and implementation of new methods for faster and safer clinical utilization to avoid potential risks [31,32,33,34]. It is important to conduct an appropriate in vitro analysis that mimics the real clinical situation as much as possible in order to assess the mechanical and biological behavior of new materials, dental implant systems, designs and dimensions, loading protocols, etc., to predict risks and unwanted clinical outcomes.

The Ti-Zr dental implants belonging to Category 3 or 2 of narrow dental implants [2] have already been proven to be successful in clinical situations with narrow alveolar ridges [35,36,37]. Four years ago, a Category 1 (diameter < 2.5 mm) new mini-implant system made from Ti-Zr Roxolid^®^ alloy was released in the dental market, having the Optiloc^®^ prosthetic connection and various transmucosal heights. The manufacturer recommended the insertion of four unsplinted MDIs in the intraforaminal region for mandibular OD support and retention. Due to the high strength and excellent biocompatibility of the Roxolid^®^ alloy, the question was raised as to whether only two MDIs can support a mandibular OD, either as single units or splinted, instead of the recommended four MDIs as single units. Therefore, this in vitro study was designed. To mimic real clinical situations, CBCT scans of a convenient subject with an edentulous atrophied mandible with narrow ridges was used for designing the models. All models represented the same mandible (except for the number of holes for MDI insertion) and were made of materials whose characteristics are similar to D2 bone density, because this is most frequently found in the anterior mandible [38]. Hao et al. reported mean values of 679.6 ± 141.67 HU density on the CBCT scans of the anterior mandible [38]. A height of 2 mm for the artificial mucosa was chosen, as it corresponds to a good clinical situation of the attached mucosa overlying a residual ridge of a denture-bearing area in patients’ mouth. The same height was also used in other studies [39,40]. All materials and procedures during the overdenture design were the same as those for a real patient, using contemporary manufacturing technology. For microstrain measurement, narrow strain gauges (1 mm) were used to minimize inaccuracies caused by anatomical factors, since they cover surfaces with curvatures. In that way, an ideal flat surface was approximated. The strain gauges were bonded as close as possible to the implants due to the assumption that the stress will be the highest where two materials of different stiffness meet (the implant and bone interface) [41]. During the bonding procedure, the models were thoroughly cleaned, and the acetate foil was used to firmly press the strain gauge on the model to ensure only a thin layer of the glue material to minimize errors due to the various thicknesses of the adhesive used [42].

The loading forces of 50, 100, and 150 N, respectively, were chosen based on reports of the minimum and maximum biting forces in individuals with conventional complete dentures, implant-supported ODs, and natural teeth [43,44,45,46,47]. The forces used represent the average forces in subjects wearing implant ODs. Bilateral loadings simulated bilateral chewing, while unilateral loadings simulated unilateral chewing. Anterior (frontal) loadings in this study were performed only with 50 N and 100 N forces because the bite forces, as well as the number of occlusal contacts, increase in the molar and premolar region compared to the incisor region [45]. However, it was also difficult to obtain higher anterior loads due to the unstable position of the metal plate on the OD’s artificial incisors during loading tasks.

The primary focus of this study was assessing strains around mini-implants supporting a mandibular OD, and strains in retromolar edentulous areas (under denture saddles) after load application at different OD positions in the three mandibular models: one unsplinted two-MDI model, one splinted two-MDI model, and one unsplinted four-MDI model. The four-MDI model represented the reference model, since four single-unit MDIs have been recommended to be placed interforaminally by a manufacturer. Furthermore, the insertion of four MDIs made of Ti90Al6V4 alloy in the intraforaminal region of the mandible, with rubber O-rings in metal housings for OD retention, has been a successful treatment option over a longer period of clinical observation [4,5,6,7,8,9,10,11,12,13,14,15,16,17]. The strains in peri-implant bone and the strains from distal edentulous areas under denture saddles are important factors predicting the long-term success of implants which support an OD, especially when knowing that a decrease in implant diameter may increase the stress transferred to the bone–implant interface [48,49]. One clinical study showed lower survival rates when two MDIs supported mandibular ODs than when two implants of standard diameter dimensions were used [11].

The results obtained in this study point out that, by increasing the loading forces, the amounts of peri-implant strains also increase in all tested models. However, peri-implant microstrains were almost two-times higher in both the unsplinted and splinted two-MDI models than in the four-MDI model (control). No significant effect of the splinting was observed on the amounts of peri-implant strains in the models with two MDIIs. Jofre et al. reported a higher amount of peri-implant marginal bone loss around two unsplinted MDIs than two splinted MDIs supporting mandibular ODs in a clinical follow-up study when Ti90Al6V4 MDIs were used [20,50]. On the contrary, our study did not record lower peri-implant microstrains in splinted mini-implants, pointing out that the splinting of two Ti-Zr mini-implants would not reduce peri-implant strains and prevent consequent marginal bone loss in cases of overloading. The same authors [50] reported less peri-implant stress in the FEA analysis in the two-MDI splinted model than in the two-MDI unsplinted (single-unit) model. The difference between their results and the results reported in this study may be due to the different retentive elements and implant materials, as Jofre et al. [20,50] used more flexible retention (rubber “O”-rings in metal housings), allowing more displacement of an OD. They also used MDIs made from Ti90Al6V4 alloy. We used the Optiloc^®^ prosthetic connection with the PEEK matrices in titanium housings (retentive force of each matrix was about 120 N) and the Roxolid^®^ alloy. The new retention system provides a higher retention than rubber O-rings and allows less OD displacement under loading conditions. Therefore, probably more loading forces were transferred to the peri-implant bone and less to the posterior edentulous area than when the O-ring retentive elements were used. The elastic characteristics of the Roxolid^®^ alloy are like the Ti-6Al-4V alloy, but with a higher tensile strength [51].

However, this study pointed out that splinting had only a significant effect on the amount of microstrains recorded from strain gauges bonded on distal edentulous areas under the OD saddles, especially when the OD was loaded with smaller forces. In most loading conditions, smaller amounts of microstrains were recorded from distal edentulous areas in the splinted model. Warin et al. [40] found that microstrain values on the retromolar edentulous areas increased as the number of MDIs increased, which is contrary to the results of this study. They assumed that the forces concentrated only at the denture contact area when the denture displacement was limited, although their retentive elements (flexible rubber “O”-rings) enabled the rotational freedom of a denture, allowing a higher amount of distal settling, contrary to the PEEK matrices used in this study [52]. All three printed models in this research were of equal dimensions, allowing strain gauge bonding in the same places in all models in the retromolar edentulous area. Posterior strain gauges were placed a little more anterior than the posterior end of the OD. This study showed that strains in the edentulous posterior region in the two-MDI splinted model were a little lower than in the two-MDI unsplinted model and could be explained by less denture subsidence distally when the bar was used. The contact surface area between the OD and the artificial mucosa and a load transfer were probably reduced by a small amount. Clinically, the finding can be interpreted thus: the splinting of two MDIs would lead to a smaller amount of posterior alveolar ridge atrophy due to the smaller amounts of denture settling and load transfer to the respective area [53].

The results of this study also revealed smaller amounts of peri-implant microstrains in the four-MDI single-unit model (control model) than in any of the two-MDI models (either unsplinted or splinted). Although the four-MDI model showed lower strains than both two-MDI models, higher strains were concentrated around distal MDIs under both bilateral and unilateral loadings, closer to the loading site. The results are contrary to Warin et al. [40], as they reported that the microstrain values around MDIs increased as the MDI number increased, with an explanation that, with increased implant numbers, more of the loading force was shared by the implants. The explanation for the lower peri-implant strains recorded in this study in the four-MDI model is that the loading forces were distributed and shared on more implants, thus reducing the strains around a single implant. The findings are similar to a recent study focused on the influence of a number of single-unit implants retaining an OD on peri-implant strains [54]. This study also showed that peri-implant strains were higher in the models with two implants, regardless of the splinting status. The highest amounts of peri-implant microstrains were recorded during unilateral loading on the loaded side in all models, corresponding to unilateral chewing. The clinical implication of this result would be to instruct patients rehabilitated with implant-supported overdentures to chew bilaterally in order to prevent the overloading of an implant on the chewing side during unilateral chewing.

Any stress on bone results in a deformation. The amount of 1000 microstrains corresponds to an approximately 0.1% deformation, which can differ a certain amount in bones with different properties [55]. When the strain increases slightly, the bone first compensates for loads by forming more bone. Bone functions without damage within the range of 50–1500 microstrains; the 1500–3000 microstrain range corresponds to mild overloads which can be repaired by reshaping and an increase of density. Repeated stress with ≥3000 microstrains increases the micro-damage and can overwhelm the reparatory mechanisms [56], especially when accounting for the fact that there is not always a linear relationship between stress and bone failure. Excessive cyclic loads can lead to defects around the already osseointegrated implants [57]. When accounting for the fact that boundaries of normal physiologic microstrains range from 1000 to 3000, it can be clearly seen that the microstrain values reported in this study did not exceed physiologic limits.

Patil et al. [58], in their 3D finite element analysis, found that two MDIs generate 68.15% more stress than two standard-sized implants. The stresses in the implants and mandible decrease with an increase in the diameter of the implants [59]. That means that precaution should be taken in cases with two mini-implants. Some factors like the diversity of bone heights in different situations can also influence the appearance of different microstrains around implants [60].

This “in vitro” study has some limitations, as it was not possible to predict a “real” situation completely. The cortical bone varies in thickness; the trabecular bone varies in architecture and density; the alveolar ridge is not of uniform bone height; the attached mucosa of a denture-bearing area is not of equal thickness and consistency in all sites, allowing unpredictable OD micro movements; and the occlusal force transfer can be multidirectional, which may complicate the stress situations. Sometimes, when natural teeth are present in the antagonistic jaw, higher loading forces may occur. Our study is based on a single CBCT scan and in vitro conditions. Therefore, further studies including more factors, such as higher loading forces, different mucosa thicknesses, and different bone morphology and density, should be undertaken to predict more clinical situations.

## 5. Conclusions

In conclusion, within the limitations of this study, the increase of loading forces induced higher peri-implant microstrains in the two-MDI models (both unsplinted and splinted) than in the four-MDI model, as well as in the respective edentulous areas under the saddles. Unilateral loading resulted in higher peri-implant microstrains than bilateral loading, especially on the loaded side. The splinting of two MDIs had no significant effects on peri-implant microstrains but led to lower microstrains in the posterior edentulous area. The findings of this study are based on an in vitro model and should be interpreted with caution when applying to clinical situations Additional studies, due to the complexity of biological systems, including clinical follow-up studies are necessary in order to validate these findings.

## Data Availability

The data are available from the corresponding author upon request.

## Supplementary Materials

The following supporting information can be downloaded at: https://www.mdpi.com/article/10.3390/biomimetics9030178/s1, Table S1: Periimplant miccrostrains obtained from straingauges around two unsplinted and two splinted Roxolid^®^ miniimplants supporting mandibular overdenture loaded bilaterally, unilaterally and anteriorly with forces of 50, 100, and 150 N applied at the second premolar and first molar area; Table S2a: MANOVA analysis with Effect Sizes (ɳ^2^) for microstrains recorded from peri-implant right- and left-side vestibular and oral straingauges (SG) in the two-MDI models dependent on Splinting Status, Loading Position and Loading Force; Table S2b: Post-hoc tests for Loading forces (the significance of the differences between peri-implant microstrains when the two MDIs were inserted dependent on different loading forces); Table S2c: Post-hoc tests for Loading position (the significance of the differences between peri-implant microstrains in the 2-MDI models depending on different Loading position on the respective overdentures); Table S2d: Comparison of microstrains obtained from periimplant straingauges dependent on splinting status (splinted vs. unsplinted); Table S3: Peri-implant miccrostrains obtained from four unsplinted Roxolid^®^ miniimplants, supporting mandibular overdenture loaded bilaterally and unilaterally with forces of 50, 100, and 150 N; Table S4a: MANOVA analysis with Effect Sizes (ɳ^2^) for microstrains recorded from peri-implant right- and left-side vestibular and oral straingauges (SG) in the four-unsplinted-MDI model dependent on Loading Position and Loading Force; Table S4b: Post-hoc tests for Loading forces in the four-MDI model (the significance of the differences between peri-implant microstrains when four MDIs were inserted dependent on different loading forces); Table S4c: Post-hoc tests for Loading position in the four-MDI model (the significance of the differences between peri-implant microstrains when four MDIs were inserted dependent on different loading positions of the respective overdentures; Table S5: Microstrains recorded from the right and left distal edentulous area (under mandibular overdenture saddles) during bilateral and unilateral (right side) loadings with 50 N, 100 N, and 150 N forces; Table S6a: MANOVA analysis with Effect Sizes (ɳ^2^) for microstrains recorded from straingauges bonded on the right and left posterior edentulous area in the two-MDI models dependent on Splinting Status, Loading Position and Loading Force; Table S6b: The significance of the differences of microstrains in the 2-MDI models recorded from strain gauges on the right and left edentulous area under denture saddles dependent on two different loading positions on the respective overdentures; Table S6c: Significance of the differences between microstrains recorded from strain gauges on the right and left edentulous area under denture saddles in the 2-MDI models dependent on Splinting status; Table S6d: Post-hoc tests for the significance of the differences between microstrains in the 2-MDI models recorded from strain gauges on the right and left edentulous area under denture saddles dependent on different Loading forces; Table S7a: Four single unit (unsplinted) MDI model; microstrains from the right and left distal edentulous area (under mandibular overdenture saddles) during bilateral and unilateral (right side) loadings with 50 N, 100 N, and 150 N forces applied at the second premolar and first molar area; Table S7b: MANOVA with Effect Sizes (ɳ^2^) for microstrains recorded from straingauges bonded on the right and left posterior edentulous area in the four-MDI model dependent on Splinting Status and Loading Position; Table S7c: The significance of the differences between microstrains recorded from the right and left edentulous area under denture saddles in the 4-MDI model when the overdenture was loaded at two different loading positions; Table S7d: The significance of the differences (post-hoc tests) between microstrains in the 4-MDI model recorded from strain gauges on the right and left edentulous area under denture saddles when the overdenture was loaded with three different loading forces.
