# Supplementary material for: Mandibular Overdenture Supported by Two or Four Unsplinted or Two Splinted Ti-Zr Mini-Implants: In Vitro Study of Peri-Implant and Edentulous Area Strains
Source: Biomimetics (Basel). 2024 Mar 15;9(3):178. doi: 10.3390/biomimetics9030178 (PMC10968521; doi:10.3390/biomimetics9030178)
Supplement: Supplementary file 1 [file biomimetics-09-00178-s001.zip › Supplementary Table S1.-2 MDI periimplant.pdf]

Table S1. Periimplant miccrostrains obtained from straingauges around two unsplinted and two splinted Roxolid® miniimplants supporting mandibular overdenture loaded bilaterally, unilaterally and anteriorly with forces of 50, 100, and 150 N applied at the second premolar and first molar area

Straingauge position	Splinting Status	Loading force	Loading position	Mean	Std. Deviation	N
Right side, Vestibular SG (position 43)	not splinted	50 N	bilateral	592.28	105.05	15
			frontal	544.89	47.55	15
			unilateral (right side)	1454.05	53.43	15
		100 N	bilateral	803.64	56.50	15
			frontal	865.43	56.74	15
			unilateral (right side)	1629.88	55.87	15
		150 N	bilateral	1051.32	63.96	15
			unilateral (right side)	1836.64	62.99	15
	splinted	50 N	bilateral	519.08	46.11	15
			frontal	510.78	56.07	15
			unilateral (right side)	1497.06	60.99	15
		100 N	bilateral	756.89	47.69	15
			frontal	956.54	59.85	15
			unilateral (right side)	1668.01	47.19	15
		150 N	bilateral	1043.47	83.42	15
			unilateral (right side)	1913.29	56.91	15
Right side, Oral SG (position 43)	not splinted	50 N	bilateral	556.04	79.90	15
			frontal	491.79	40.13	15
			unilateral (right side)	1334.25	73.37	15
		100 N	bilateral	668.82	80.65	15
			frontal	679.00	57.27	15
			unilateral (right side)	1546.38	56.81	15
		150 N	bilateral	865.50	68.22	15
			unilateral (right side)	1726.63	81.28	15
	splinted	50 N	bilateral	488.66	69.38	15
			frontal	419.20	54.35	15
			unilateral (right side)	1339.14	345.29	15
		100 N	bilateral	529.87	93.56	15
			frontal	779.63	54.67	15
			unilateral (right side)	1638.61	64.12	15
		150 N	bilateral	782.08	79.63	15
			unilateral (right side)	1867.68	47.47	15

Left side, Vestibular SG (position 43)	not splinted	50 N	bilateral	569.83	83.93	15
			frontal	551.59	82.63	15
			unilateral (right side)	1039.17	61.23	15
		100 N	bilateral	767.61	60.86	15
			frontal	829.93	53.55	15
			unilateral (right side)	1294.77	76.91	15
		150 N	bilateral	1071.21	53.40	15
			unilateral (right side)	1444.48	54.57	15
	splinted	50 N	bilateral	614.36	36.77	15
			frontal	541.16	48.98	15
			unilateral (right side)	1088.36	129.26	15
		100 N	bilateral	765.03	55.92	15
			frontal	941.83	73.84	15
			unilateral (right side)	1368.32	77.61	15
		150 N	bilateral	1044.04	68.78	15
			unilateral (right side)	1670.83	47.94	15
Left side, oral SG (position 44)	not splinted	50 N	bilateral	465.05	20.22	15
			frontal	504.75	64.34	15
			unilateral (right side)	994.98	57.58	15
		100 N	bilateral	690.80	64.17	15
			frontal	673.78	55.69	15
			unilateral (right side)	1267.99	34.62	15
		150 N	bilateral	824.68	78.46	15
			unilateral (right side)	1332.27	80.56	15
	splinted	50 N	bilateral	412.04	63.65	15
			frontal	415.53	49.51	15
			unilateral (right side)	935.04	41.87	15
		100 N	bilateral	518.49	50.68	15
			frontal	769.51	49.51	15
			unilateral (right side)	1432.29	67.96	15
		150 N	bilateral	773.98	59.09	15
			unilateral (right side)	1588.15	78.91	15
