# Supplementary material for: Mandibular Overdenture Supported by Two or Four Unsplinted or Two Splinted Ti-Zr Mini-Implants: In Vitro Study of Peri-Implant and Edentulous Area Strains
Source: Biomimetics (Basel). 2024 Mar 15;9(3):178. doi: 10.3390/biomimetics9030178 (PMC10968521; doi:10.3390/biomimetics9030178)
Supplement: Supplementary file 1 [file biomimetics-09-00178-s001.zip › Supplementary Table S2 a-d. MANOVA 2 MDI by loading position, loading force and splinting status and post-hoc tests.pdf]

Supplementary Table S2a. MANOVA analysis with Effect Sizes (η²) for microstrains recorded from peri-implant right- and left-side vestibular and oral straingauges (SG) in the two-MDI models dependent on Splinting Status, Loading Position and Loading Force

Source	Dependent Variable	Type III Sum of Squares	df	Mean Square	F	*P*	Partial Eta Squared (η²)
Corrected Model	Right side, vestibular SG (position 43) -	54415241.609[a]	15	3627682.774	951.256	<0.001**	0.985
	Right side, oral SG (position 43)	57163072.918[b]	15	3810871.528	322.467	<0.001**	0.956
	Left side, vestibular SG (position 33)	26452545.532[c]	15	1763503.035	361.612	<0.001**	0.960
	Left side, oral SG (position 33)	32199441.219[d]	15	2146629.415	607.444	<0.001**	0.976
Intercept	Right side, vestibular SG (position 43) -	279342740.508	1	279342740.508	73249.669	<0.001**	0.997
	Right side, oral SG (position 43)	219803411.168	1	219803411.168	18599.255	<0.001**	0.988
	Left side, vestibular SG (position 33)	222763952.059	1	222763952.059	45678.461	<0.001**	0.995
	Left side, oral SG (position 33)	167528902.899	1	167528902.899	47406.630	<0.001**	0.995
Splinting status	Right side, vestibular SG (position 43) -	12959.432	1	12959.432	3.398	0.067 N.S.	0.015
	Right side, oral SG (position 43)	214.732	1	214.732	.018	0.893 N.S.	0.000
	Left side, vestibular SG (position 33)	218689.783	1	218689.783	44.843	<0.001**	0.167
	Left side, oral SG (position 33)	22296.053	1	22296.053	6.309	0.013 N.S.	0.027
Loading Position	Right side, vestibular SG (position 43) -	40483827.534	2	20241913.767	5307.865	<0.001**	0.979
	Right side, oral SG (position 43)	45732435.418	2	22866217.709	1934.886	<0.001**	0.945
	Left side, vestibular SG (position 33)	13787562.741	2	6893781.371	1413.592	<0.001**	0.927
	Left side, oral SG (position 33)	21153152.193	2	10576576.096	2992.915	<0.001**	0.964
Loading Force	Right side, vestibular SG (position 43) -	7841291.551	2	3920645.776	1028.078	<0.001**	0.902
	Right side, oral SG (position 43)	5386215.395	2	2693107.698	227.885	<0.001**	0.670
	Left side, vestibular SG (position 33)	8506402.954	2	4253201.477	872.133	<0.001**	0.886
	Left side, oral SG (position 33)	6671060.757	2	3335530.378	943.874	<0.001**	0.894
Splinting status * Loading Position	Right side, vestibular SG (position 43) -	116552.968	2	58276.484	15.281	<0.001**	0.120
	Right side, oral SG (position 43)	365442.171	2	182721.085	15.461	<0.001**	0.121
	Left side, vestibular SG (position 33)	140621.745	2	70310.872	14.417	<0.001**	0.114
	Left side, oral SG (position 33)	517253.784	2	258626.892	73.185	<0.001**	0.395
Splinting status * Loading Force	Right side, vestibular SG (position 43) -	46384.344	2	23192.172	6.081	0.003**	0.052
	Right side, oral SG (position 43)	76138.922	2	38069.461	3.221	0.042*	0.028
	Left side, vestibular SG (position 33)	46522.564	2	23261.282	4.770	0.009**	0.041
	Left side, oral SG (position 33)	281474.986	2	140737.493	39.825	<0.001**	0.262
Loading Position * Loading Force	Right side, vestibular SG (position 43) -	403305.728	3	134435.243	35.252	<0.001**	0.321
	Right side, oral SG (position 43)	403625.563	3	134541.854	11.385	<0.001**	0.132
	Left side, vestibular SG (position 33)	209478.152	3	69826.051	14.318	<0.001**	0.161
	Left side, oral SG (position 33)	367992.327	3	122664.109	34.711	<0.001**	0.317
Splinting status * Loading Position * Loading Force	Right side, vestibular SG (position 43) -	35217.074	3	11739.025	3.078	0.028*	0.040
	Right side, oral SG (position 43)	128914.192	3	42971.397	3.636	0.014*Tab	0.046
	Left side, vestibular SG (position 33)	167819.168	3	55939.723	11.471	<0.001**	0.133
	Left side, oral SG (position 33)	315403.251	3	105134.417	29.750	<0.001**	0.285

Error	Right side, vestibular SG (position 43) -	854239.676	224	3813.570
	Right side, oral SG (position 43)	2647200.842	224	11817.861
	Left side, vestibular SG (position 33)	1092399.439	224	4876.783
	Left side, oral SG (position 33)	791587.040	224	3533.871
Total	Right side, vestibular SG (position 43) -	347098051.116	240	
	Right side, oral SG (position 43)	291285531.204	240	
	Left side, vestibular SG (position 33)	255768333.423	240	
	left side, oral SG (position 33)	206373682.201	240	
Corrected Total	Right side, vestibular SG (position 43) -	55269481.285	239	
	Right side, oral SG (position 43)	59810273.760	239	
	Left side, vestibular SG (position 33)	27544944.971	239	
	left side, oral SG (position 33)	32991028.259	239	

a. R Squared = .985 (Adjusted R Squared = .984)

b. R Squared = .956 (Adjusted R Squared = .953)

c. R Squared = .960 (Adjusted R Squared = .958)

d. R Squared = .976 (Adjusted R Squared = .974)

SG=straingauge; df=Degree of Freedom; **=significant at p<0.01, *=significant at p<0.05, N.S.= not significant (p>0.05)

Supplementary Table S2b. Post-hoc tests for Loading forces (the significance of the differences between peri-implant microstrains when the two MDIs were inserted dependent on different loading forces)

Post-hoc tests: Loading forces (2-MDIs)

Dependent Variable	Loading force		Mean Difference (I-J)	SE	*P*.[d]	95% Confidence Interval for Difference[d]	
						Lower Bound	Upper Bound
Right side, vestibular SG (position 43) -	50 N	100 N	-260.374[*]	9.21	<0.001**	-282.52	-238.23
		150 N	-608.157[*,b]	10.29	<0.001**	-632.92	-583.40
	100 N	50 N	260.374[*]	9.21	<0.001**	238.23	282.52
		150 N	-347.783[*,b]	10.29	<0.001**	-372.54	-323.02
	150 N	50 N	608.157[*,c]	10.29	<0.001**	583.40	632.92
		100 N	347.783[*,c]	10.29	<0.001**	323.02	372.54
Right side, oral SG (position 43)	50 N	100 N	-202.206[*]	16.21	<0.001**	-241.19	-163.22
		150 N	-538.961[*,b]	18.12	<0.001**	-582.55	-495.37
	100 N	50 N	202.206[*]	16.21	<0.001**	163.22	241.19
		150 N	-336.755[*,b]	18.12	<0.001**	-380.34	-293.17
	150 N	50 N	538.961[*,c]	18.12	<0.001**	495.37	582.55
		100 N	336.755[*,c]	18.12	<0.001**	293.17	380.34
Left side, vestibular SG (position 33)	50 N	100 N	-260.504[*]	10.41	<0.001**	-285.55	-235.46
		150 N	-573.561[*,b]	11.64	<0.001**	-601.56	-545.56
	100 N	50 N	260.504[*]	10.41	<0.001**	235.46	285.55

		150 N	-313.057*,b	11.64	<0.001**	-341.06	-285.06
	150 N	50 N	573.561*,c	11.64	<0.001**	545.56	601.56
		100 N	313.057*,c	11.64	<0.001**	285.06	341.06
left side, oral SG (position 33)	50 N	100 N	-270.915*	8.86	<0.001**	-292.23	-249.60
		150 N	-508.540*,b	9.91	<0.001**	-532.38	-484.71
	100 N	50 N	270.915*	8.86	<0.001**	249.60	292.23
		150 N	-237.626*,b	9.91	<0.001**	-261.46	-213.79
	150 N	50 N	508.540*,c	9.91	<0.001**	484.71	532.38
		100 N	237.626*,c	9.91	<0.001**	213.79	261.46

Based on estimated marginal means

*. The mean difference is significant at the .05 level.

b. An estimate of the modified population marginal mean (J).

c. An estimate of the modified population marginal mean (I).

d. Adjustment for multiple comparisons: Sidak.

SE=standard error; *P*=significance, **=p<0.01

Supplementary Table S2c. Post-hoc tests for Loading position (the significance of the differences between peri-implant microstrains in the 2-MDI models depending on different Loading position on the respective overdentures)

Post-hos tests: Loading position (2 MDIs)

Dependent Variable	Loading position		Mean Difference (I-J)	SE	P.[d]	95% Confidence Interval for Difference[d]	
						Lower Bound	Upper Bound
Right side, vestibular SG (position 43) -	bilateral	frontal	75.040*,b	10.29	<0.001**	50.28	99.80
		unilateral (right side)	-872.042*	9.21	<0.001**	-894.19	-849.90
	frontal	bilateral	-75.040*,c	10.29	<0.001**	-99.80	-50.28
		unilateral (right side)	-947.081*,c	10.29	<0.001**	-971.84	-922.32
	unilateral (right side)	bilateral	872.042*	9.21	<0.001**	849.90	894.19
		frontal	947.081*,b	10.29	<0.001**	922.32	971.84
Right side, oral SG (position 43)	bilateral	frontal	56.088*,b	18.12	0.007**	12.50	99.68
		unilateral (right side)	-926.953*	16.21	<0.001**	-965.94	-887.97
	frontal	bilateral	-56.088*,c	18.12	0.007**	-99.68	-12.50
		unilateral (right side)	-983.041*,c	18.12	<0.001**	-1026.63	-939.45
	unilateral (right side)	bilateral	926.953*	16.21	<0.001**	887.97	965.94
		frontal	983.041*,b	18.12	<0.001**	939.45	1026.63
Left side, vestibular SG (position 33)	bilateral	frontal	89.215*,b	11.64	<0.001**	61.22	117.22
		unilateral (right side)	-512.312*	10.41	<0.001**	-537.36	-487.27
	frontal	bilateral	-89.215*,c	11.64	<0.001**	-117.22	-61.22
		unilateral (right side)	-601.527*,c	11.64	<0.001**	-629.53	-573.53
	unilateral (right side)	bilateral	512.312*	10.41	<0.001**	487.27	537.36
		frontal	601.527*,b	11.64	<0.001**	573.53	629.53

left side, oral SG (position 33)	bilateral	frontal	23.284[b]	9.91	.058	-0.55	47.12
		unilateral (right side)	-644.277[*]	8.86	<0.001**	-665.60	-622.96
	frontal	bilateral	-23.284[c]	9.91	.058	-47.12	0.55
		unilateral (right side)	-667.561[*,c]	9.91	<0.001**	-691.40	-643.73
	unilateral (right side)	bilateral	644.277[*]	8.86	<0.001**	622.96	665.60
		frontal	667.561[*,b]	9.91	<0.001**	643.73	691.40

Based on estimated marginal means

*. The mean difference is significant at the .05 level.

b. An estimate of the modified population marginal mean (J).

c. An estimate of the modified population marginal mean (I).

d. Adjustment for multiple comparisons: Sidak.

SE=standard error; *P*=significance, p value; **p<0.01

Supplementary Table S2d. Comparison of microstrains obtained from periimplant straingauges dependent on splinting status (splinted vs. unsplinted)

Splinting status

Dependent Variable			Mean Difference (I-J)	SE	P^d	95% Confidence Interval for Difference[d]	
						Lower Bound	Upper Bound
Right side, vestibular SG (position 43) -	not splinted	splinted	-10.872[a,b]	7.97	0.17 N.S.	-26.58	4.84
	splinted	not splinted	10.872[a,b]	7.97	0.17 N.S.	-4.84	26.58
Right side, oral SG (position 43)	not splinted	splinted	2.942[a,b]	14.03	0.83 N.S.	-24.71	30.60
	splinted	not splinted	-2.942[a,b]	14.03	0.83 N.S.	-30.60	24.71
Left side, vestibular SG (position 33)	not splinted	splinted	-58.169[a,b,*]	9.02	<0.01	-75.93	-40.40
	splinted	not splinted	58.169[a,b,*]	9.02	<0.01	40.40	75.93
left side, oral SG (position 33)	not splinted	splinted	-11.342[a,b]	7.67	0.14 N.S.	-26.47	3.78
	splinted	not splinted	11.342[a,b]	7.67	0.14 N.S.	-3.78	26.47

Based on estimated marginal means

*. The mean difference is significant at the .05 level.

a. An estimate of the modified population marginal mean (I).

b. An estimate of the modified population marginal mean (J).

d. Adjustment for multiple comparisons: Sidak.

SE=standard error; *P*=significance, p value; N.S.=not significant
