# Supplementary material for: Mandibular Overdenture Supported by Two or Four Unsplinted or Two Splinted Ti-Zr Mini-Implants: In Vitro Study of Peri-Implant and Edentulous Area Strains
Source: Biomimetics (Basel). 2024 Mar 15;9(3):178. doi: 10.3390/biomimetics9030178 (PMC10968521; doi:10.3390/biomimetics9030178)
Supplement: Supplementary file 1 [file biomimetics-09-00178-s001.zip › supplementary Table S3, 4 MDI oko implantata.pdf]

Supplementary Table S3. Peri-implant miccrostrains obtained from four unsplinted Roxolid® miniimplants, supporting mandibular overdenture loaded bilaterally and unilaterally with forces of 50, 100, and 150 N

Straingauge position	Loading force	Loading position	Mean	Std. Deviation	N
Right side, vestibular SG, site 44	50 N	bilateral	473.25	83.98	15
		frontal	402.72	41.17	15
		unilateral (right side)	603.54	81.27	15
	100 N	bilateral	677.70	74.17	15
		frontal	485.08	44.68	15
		unilateral (right side)	793.13	41.78	15
	150 N	bilateral	848.72	61.22	15
		unilateral (right side)	989.64	63.08	15
Right side, oral SG, position 44	50 N	bilateral	394.63	70.15	15
		frontal	268.74	34.99	15
		unilateral (right side)	529.77	77.02	15
	100 N	bilateral	583.32	49.45	15
		frontal	419.18	51.03	15
		unilateral (right side)	695.88	59.33	15
	150 N	bilateral	803.51	40.72	15
		unilateral (right side)	904.48	54.12	15
Left side, vestibular SG, position 34	50 N	bilateral	284.89	38.62	15
		frontal	490.11	32.97	15
		unilateral (right side)	400.39	42.72	15
	100 N	bilateral	413.32	55.07	15
		frontal	611.32	56.61	15
		unilateral (right side)	494.16	72.07	15
	150 N	bilateral	645.28	43.36	15
		unilateral (right side)	606.80	59.34	15
Left side, oral SG, position 34	50 N	bilateral	314.14	35.72	15
		frontal	380.28	40.34	15
		unilateral (right side)	413.90	41.61	15
	100 N	bilateral	410.17	71.29	15
		frontal	501.06	39.01	15
		unilateral (right side)	494.59	33.30	15
	150 N	bilateral	606.92	48.84	15
		unilateral (right side)	599.60	84.99	15
Right side, vestibular SG, site 42	50 N	bilateral	339.27	57.90	15
		frontal	518.45	44.32	15
		unilateral (right side)	500.58	31.07	15
	100 N	bilateral	392.59	58.66	15

		frontal	624.88	57.56	15
		unilateral (right side)	691.10	56.10	15
	150 N	bilateral	638.56	61.63	15
		unilateral (right side)	805.30	31.86	15
**Right side, oral SG, position 42**	50 N	bilateral	296.57	45.76	15
		frontal	387.69	51.89	15
		unilateral (right side)	481.10	57.13	15
	100 N	bilateral	377.74	32.23	15
		frontal	500.19	57.17	15
		unilateral (right side)	609.00	38.59	15
	150 N	bilateral	603.45	45.85	15
		unilateral (right side)	709.65	54.90	15
**Left side, vestibular SG, site 32**	50 N	bilateral	413.11	50.68	15
		frontal	434.74	35.86	15
		unilateral (right side)	304.33	34.03	15
	100 N	bilateral	723.16	67.40	15
		frontal	491.69	44.42	15
		unilateral (right side)	427.27	52.18	15
	150 N	bilateral	864.48	49.74	15
		unilateral (right side)	458.80	28.42	15
**Left side, oral SG, position 32**	50 N	bilateral	386.54	37.05	15
		frontal	303.89	36.11	15
		unilateral (right side)	305.71	25.90	15
	100 N	bilateral	576.33	46.51	15
		frontal	393.88	39.22	15
		unilateral (right side)	387.17	56.51	15
	150 N	bilateral	790.48	31.02	15
		unilateral (right side)	546.38	72.24	15
