# Supplementary material for: Mandibular Overdenture Supported by Two or Four Unsplinted or Two Splinted Ti-Zr Mini-Implants: In Vitro Study of Peri-Implant and Edentulous Area Strains
Source: Biomimetics (Basel). 2024 Mar 15;9(3):178. doi: 10.3390/biomimetics9030178 (PMC10968521; doi:10.3390/biomimetics9030178)
Supplement: Supplementary file 1 [file biomimetics-09-00178-s001.zip › Supplementary Table S4a-c. MANOVA 4 MDI i post-hoc.pdf]

Supplementary Table S4a. MANOVA analysis with Effect Sizes (η²) for microstrains recorded from peri-implant right- and left-side vestibular and oral straingauges (SG) in the four-unsplinted-MDI model dependent on Loading Position and Loading Force

Source	Dependent Variable	Type III Sum of Squares	df	Mean Square	F	*P*	Partial Eta Squared
Corrected Model	Right side, vestibular SG (44)	4457441.833[a]	7	636777.405	157.604	<0.01**	0.908
	Right side, oral SG (44)	4921652.930[b]	7	703093.276	222.892	<0.01**	0.933
	Left side, vestibular SG (34)	1625766.677[c]	7	232252.382	87.487	<0.01**	0.845
	left side, oral SG (34)	1139809.116[d]	7	162829.874	59.327	<0.01**	0.788
	Right side, vestibular SG (42)	2544374.526[e]	7	363482.075	138.486	<0.01**	0.896
	Right side, oral SG (42)	2035312.586[f]	7	290758.941	122.666	<0.01**	0.885
	Left side, vestibular SG (32)	3571091.808[g]	7	510155.973	232.777	<0.01**	0.936
	Left side, oral SG (32)	2901662.012[h]	7	414523.145	201.907	<0.01**	0.927
Intercept	Right side, vestibular SG (44)	50824425.773	1	50824425.773	12579.198	<0.01**	0.991
	Right side, oral SG (44)	38781610.552	1	38781610.552	12294.380	<0.01**	0.991
	Left side, vestibular SG (34)	30283049.942	1	30283049.942	11407.348	<0.01**	0.990
	Left side, oral SG (34)	26193662.387	1	26193662.387	9543.607	<0.01**	0.988
	Right side, vestibular SG (42)	38873343.652	1	38873343.652	14810.666	<0.01**	0.992
	Right side, oral SG (42)	29611207.274	1	29611207.274	12492.388	<0.01**	0.991
	Left side, vestibular SG (32)	31711580.061	1	31711580.061	14469.575	<0.01**	0.992
	Left side, oral SG (32)	25572152.952	1	25572152.952	12455.740	<0.01**	0.991
Loading force	Right side, vestibular SG (44)	2160929.572	2	1080464.786	267.418	<0.01**	0.827
	Right side, oral SG (44)	2475527.142	2	1237763.571	392.390	<0.01**	0.875
	Left side, vestibular SG (34)	1333161.784	2	666580.892	251.095	<0.01**	0.818
	Left side, oral SG (34)	982218.558	2	491109.279	178.935	<0.01**	0.762
	Right side, vestibular SG (42)	1468750.318	2	734375.159	279.795	<0.01**	0.833
	Right side, oral SG (42)	1186864.621	2	593432.310	250.357	<0.01**	0.817
	Left side, vestibular SG (32)	1358060.531	2	679030.265	309.833	<0.01**	0.847
	Left side, oral SG (32)	1621541.499	2	810770.750	394.912	<0.01**	0.876
Loading position	Right side, vestibular SG (44)	1118786.996	2	559393.498	138.452	<0.01**	0.712
	Right side, oral SG (44)	1160408.239	2	580204.120	183.934	<0.01**	0.767
	Left side, vestibular SG (34)	527578.159	2	263789.080	99.367	<0.01**	0.640
	Left side, oral SG (34)	99293.517	2	49646.759	18.089	<0.01**	0.244
	Right side, vestibular SG (42)	1146264.026	2	573132.013	218.362	<0.01**	0.796
	Right side, oral SG (42)	681333.790	2	340666.895	143.721	<0.01**	0.720
	Left side, vestibular SG (32)	1641990.727	2	820995.364	374.609	<0.01**	0.870
	Left side, oral SG (32)	745367.499	2	372683.750	181.528	<0.01**	0.764
Loading force * Loading position	Right side, vestibular SG (44)	68190.785	3	22730.262	5.626	<0.01**	0.131
	Right side, oral SG (44)	8162.678	3	2720.893	.863	0.463	0.023
	Left side, vestibular SG (34)	98373.104	3	32791.035	12.352	<0.01**	0.249
	Left side, oral SG (34)	55553.307	3	18517.769	6.747	<0.01**	0.153
	Right side, vestibular SG (42)	91742.233	3	30580.744	11.651	<0.01**	0.238

	Right side, oral SG (42)	60222.428	3	20074.143	8.469	<0.01**	0.185
	Left side, vestibular SG (32)	465304.691	3	155101.564	70.771	<0.01**	0.655
	Left side, oral SG (32)	113931.213	3	37977.071	18.498	<0.01**	0.331
Error	Right side, vestibular SG (44)	452519.755	112	4040.355			
	Right side, oral SG (44)	353294.796	112	3154.418			
	Left side, vestibular SG (34)	297326.051	112	2654.697			
	Left side, oral SG (34)	307398.478	112	2744.629			
	Right side, vestibular SG (42)	293964.800	112	2624.686			
	Right side, oral SG (42)	265478.074	112	2370.340			
	Left side, vestibular SG (32)	245459.669	112	2191.604			
	Left side, oral SG (32)	229940.655	112	2053.042			
Total	Right side, vestibular SG (44)	57058821.899	120				
	Right side, oral SG (44)	44941623.272	120				
	Left side, vestibular SG (34)	31122461.544	120				
	Left side, oral SG (34)	27403245.926	120				
	Right side, vestibular SG (42)	40988407.272	120				
	Right side, oral SG (42)	31783753.708	120				
	Left side, vestibular SG (32)	35606458.273	120				
	Left side, oral SG (32)	28667223.343	120				
Corrected Total	Right side, vestibular SG (44)	4909961.588	119				
	Right side, oral SG (44)	5274947.726	119				
	Left side, vestibular SG (34)	1923092.728	119				
	Left side, oral SG (34)	1447207.594	119				
	Right side, vestibular SG (42)	2838339.326	119				
	Right side, oral SG (42)	2300790.659	119				
	Left side, vestibular SG (32)	3816551.477	119				
	Left side, oral SG (32)	3131602.667	119				

a. R Squared = .908 (Adjusted R Squared = .902)

b. R Squared = .933 (Adjusted R Squared = .929)

c. R Squared = .845 (Adjusted R Squared = .836)

d. R Squared = .788 (Adjusted R Squared = .774)

e. R Squared = .896 (Adjusted R Squared = .890)

f. R Squared = .885 (Adjusted R Squared = .877)

g. R Squared = .936 (Adjusted R Squared = .932)

h. R Squared = .927 (Adjusted R Squared = .922)

df=Degree of freedom; *P*=Level of significance

Supplementary Table S4 b. Post-hoc tests for Loading forces in the four-MDI model (the significance of the differences between peri-implant microstrains when four MDIs were inserted dependent on different loading forces)

Four MDI-model (Unsplinted MDIs) Post hoc test: loading forces

Dependent Variable	Loading force		Mean Difference (I-J)	SE	*P* [d]	95% Confidence Interval for Difference[d] Lower Bound	Upper Bound
Right side, vestibular SG (position 44) -	50 N	100 N	-158.797*	13.40	<0.01**	-191.28	-126.31
		150 N	-426.007*,b	14.98	<0.01**	-462.32	-389.69
	100 N	50 N	158.797*	13.40	<0.01**	126.31	191.28
		150 N	-267.210*,b	14.98	<0.01**	-303.53	-230.89
	150 N	50 N	426.007*,c	14.98	<0.01**	389.69	462.32
		100 N	267.210*,c	14.98	<0.01**	230.89	303.53
Right side, oral SG (position 44)	50 N	100 N	-168.415*	11.84	<0.01**	-197.12	-139.71
		150 N	-456.283*,b	13.24	<0.01**	-488.37	-424.19
	100 N	50 N	168.415*	11.84	<0.01**	139.71	197.12
		150 N	-287.868*,b	13.24	<0.01**	-319.96	-255.78
	150 N	50 N	456.283*,c	13.24	<0.01**	424.19	488.37
		100 N	287.868*,c	13.24	<0.01**	255.78	319.96
Left side, vestibular SG (position 34)	50 N	100 N	-114.474*	10.86	<0.01**	-140.80	-88.14
		150 N	-234.245*,b	12.14	<0.01**	-263.68	-204.81
	100 N	50 N	114.474*	10.86	<0.01**	88.14	140.80
		150 N	-119.771*,b	12.14	<0.01**	-149.21	-90.33
	150 N	50 N	234.245*,c	12.14	<0.01**	204.81	263.68
		100 N	119.771*,c	12.14	<0.01**	90.33	149.21
Left side, oral SG (position 34)	50 N	100 N	-99.168*	11.04	<0.01**	-125.94	-72.40
		150 N	-233.817*,b	12.35	<0.01**	-263.75	-203.89
	100 N	50 N	99.168*	11.04	<0.01**	72.40	125.94
		150 N	-134.649*,b	12.35	<0.01**	-164.58	-104.72
	150 N	50 N	233.817*,c	12.35	<0.01**	203.89	263.75
		100 N	134.649*,c	12.35	<0.01**	104.72	164.58
Right side, vestibular SG (position 42)	50 N	100 N	-116.753*	10.80	<0.01**	-142.93	-90.57
		150 N	-269.160*,b	12.08	<0.01**	-298.43	-239.89
	100 N	50 N	116.753*	10.80	<0.01**	90.57	142.93
		150 N	-152.406*,b	12.08	<0.01**	-181.68	-123.14
	150 N	50 N	269.160*,c	12.08	<0.01**	239.89	298.43
		100 N	152.406*,c	12.08	<0.01**	123.14	181.68
Right side, oral SG (position 42)	50 N	100 N	-107.185*	10.26	<0.01**	-132.06	-82.31
		150 N	-268.096*,b	11.48	<0.01**	-295.91	-240.28
	100 N	50 N	107.185*	10.26	<0.01**	82.31	132.06
		150 N	-160.911*,b	11.48	<0.01**	-188.73	-133.10
	150 N	50 N	268.096*,c	11.48	<0.01**	240.28	295.91
		100 N	160.911*,c	11.48	<0.01**	133.10	188.73
Left side, vestibular SG (position 32)	50 N	100 N	-163.314*	9.87	<0.01**	-187.24	-139.39
		150 N	-277.581*,b	11.03	<0.01**	-304.33	-250.83
	100 N	50 N	163.314*	9.87	<0.01**	139.39	187.24

		150 N	-114.266[*,b]	11.03	<0.01**	-141.01	-87.52
	150 N	50 N	277.581[*,c]	11.03	<0.01**	250.83	304.33
		100 N	114.266[*,c]	11.03	<0.01**	87.52	141.01
Left side, oral SG (position 32)	50 N	100 N	-120.413[*]	9.55	<0.01**	-143.57	-97.26
		150 N	-336.381[*,b]	10.68	<0.01**	-362.27	-310.49
	100 N	50 N	120.413[*]	9.55	<0.01**	97.26	143.57
		150 N	-215.969[*,b]	10.68	<0.01**	-241.86	-190.08
	150 N	50 N	336.381[*,c]	10.68	<0.01**	310.49	362.27
		100 N	215.969[*,c]	10.68	<0.01**	190.08	241.86

Based on estimated marginal means
*. The mean difference is significant at the .05 level.
b. An estimate of the modified population marginal mean (J).
c. An estimate of the modified population marginal mean (I).
d. Adjustment for multiple comparisons: Sidak.
SE= Standard Error; P=significance, p value; **=p<0.01

Supplementary Table S4 c. Post-hoc tests for Loading position in the four-MDI model (the significance of the differences between peri-implant microstrains when four MDIs were inserted dependent on different loading positions of the respective overdentures

Four MDI-model (Unsplinted MDIs) Post hoc test: loading position

Dependent Variable			Mean Difference (I-J)	SE	P[d]	95% Confidence Interval for Difference[d] Lower Bound	Upper Bound
Right side, vestibular SG (position 44) -	bilateral	frontal	222.656[*,b]	14.98	<0.01**	186.34	258.97
		unilateral (right side)	-128.884[*]	13.40	<0.01**	-161.37	-96.40
	frontal	bilateral	-222.656[*,c]	14.98	<0.01**	-258.97	-186.34
		unilateral (right side)	-351.540[*,c]	14.98	<0.01**	-387.86	-315.22
	unilateral (right side)	bilateral	128.884[*]	13.40	<0.01**	96.40	161.37
		frontal	351.540[*,b]	14.98	<0.01**	315.22	387.86
Right side, oral SG (position 44)	bilateral	frontal	249.856[*,b]	13.24	<0.01**	217.77	281.94
		unilateral (right side)	-116.227[*]	11.84	<0.01**	-144.93	-87.53
	frontal	bilateral	-249.856[*,c]	13.24	<0.01**	-281.94	-217.77
		unilateral (right side)	-366.083[*,c]	13.24	<0.01**	-398.17	-333.99
	unilateral (right side)	bilateral	116.227[*]	11.84	<0.01**	87.53	144.93
		frontal	366.083[*,b]	13.24	<0.01**	333.99	398.17
Left side, vestibular SG (position 34)	bilateral	frontal	-102.885[*,b]	12.14	<0.01**	-132.32	-73.45
		unilateral (right side)	-52.618[*]	10.86	<0.01**	-78.95	-26.29
	frontal	bilateral	102.885[*,c]	12.14	<0.01**	73.45	132.32
		unilateral (right side)	50.267[*,c]	12.14	<0.01**	20.83	79.70
	unilateral (right side)	bilateral	52.618[*]	10.86	<0.01**	26.29	78.95
		frontal	-50.267[*,b]	12.14	<0.01**	-79.70	-20.83
	bilateral	frontal	3.069[b]	12.35	0.992	-26.86	33.00

		unilateral (right side)	-58.955[*]	11.04	<0.01**	-85.73	-32.18
Left side, oral SG (position 34)	frontal	bilateral	-3.069[c]	12.35	0.992	-33.00	26.86
		unilateral (right side)	-62.024[*,c]	12.35	<0.01**	-91.96	-32.09
	unilateral (right side)	bilateral	58.955[*]	11.04	<0.01**	32.18	85.73
		frontal	62.024[*,b]	12.35	<0.01**	32.09	91.96
Right side, vestibular SG (position 42)	bilateral	frontal	-114.861[*,b]	12.08	<0.01**	-144.13	-85.59
		unilateral (right side)	-208.852[*]	10.80	<0.01**	-235.03	-182.67
	frontal	bilateral	114.861[*,c]	12.08	<0.01**	85.59	144.13
		unilateral (right side)	-93.991[*,c]	12.08	<0.01**	-123.26	-64.72
	unilateral (right side)	bilateral	208.852[*]	10.80	<0.01**	182.67	235.03
		frontal	93.991[*,b]	12.08	<0.01**	64.72	123.26
Right side, oral SG (position 42)	bilateral	frontal	-18.020[b]	11.48	0.317	-45.84	9.80
		unilateral (right side)	-173.995[*]	10.26	<0.01**	-198.87	-149.12
	frontal	bilateral	18.020[c]	11.48	0.317	-9.80	45.84
		unilateral (right side)	-155.975[*,c]	11.48	<0.01**	-183.79	-128.16
	unilateral (right side)	bilateral	173.995[*]	10.26	<0.01**	149.12	198.87
		frontal	155.975[*,b]	11.48	<0.01**	128.16	183.79
Left side, vestibular SG (position 32)	bilateral	frontal	203.703[*,b]	11.03	<0.01**	176.96	230.45
		unilateral (right side)	270.120[*]	9.87	<0.01**	246.20	294.04
	frontal	bilateral	-203.703[*,c]	11.03	<0.01**	-230.45	-176.96
		unilateral (right side)	66.417[*,c]	11.03	<0.01**	39.67	93.16
	unilateral (right side)	bilateral	-270.120[*]	9.87	<0.01**	-294.04	-246.20
		frontal	-66.417[*,b]	11.03	<0.01**	-93.16	-39.67
Left side, oral SG (position 32)	bilateral	frontal	235.565[*,b]	10.68	<0.01**	209.68	261.45
		unilateral (right side)	171.365[*]	9.55	<0.01**	148.21	194.52
	frontal	bilateral	-235.565[*,c]	10.68	<0.01**	-261.45	-209.68
		unilateral (right side)	-64.200[*,c]	10.68	<0.01**	-90.09	-38.31
	unilateral (right side)	bilateral	-171.365[*]	9.55	<0.01**	-194.52	-148.21
		frontal	64.200[*,b]	10.68	<0.01**	38.31	90.09

Based on estimated marginal means
*. The mean difference is significant at the .05 level.
b. An estimate of the modified population marginal mean (J).
c. An estimate of the modified population marginal mean (I).
d. Adjustment for multiple comparisons: Sidak.
SE= Standard Error; P=significance, p value; **=p<0.01
