# Supplementary material for: Mandibular Overdenture Supported by Two or Four Unsplinted or Two Splinted Ti-Zr Mini-Implants: In Vitro Study of Peri-Implant and Edentulous Area Strains
Source: Biomimetics (Basel). 2024 Mar 15;9(3):178. doi: 10.3390/biomimetics9030178 (PMC10968521; doi:10.3390/biomimetics9030178)
Supplement: Supplementary file 1 [file biomimetics-09-00178-s001.zip › Supplementary Table S5 -2MDI distalno slijeganje descriptive (splintani i nesplintani).pdf]

Supplementary Table S5. Microstrains recorded from the right and left distal edentulous area (under mandibular overdenture saddles) during bilateral and unilateral (right side) loadings with 50 N, 100 N, and 150 N forces

Straingauge position	Loading position	Splinting status	Loading force	Mean	Std. Deviation	N
Right distal edentulous area	Bilateral loading	not splinted	50 N	394.7289	66.99021	15
			100 N	616.4494	82.75862	15
			150 N	1054.9375	113.06787	15
		splinted	50 N	455.7541	50.20826	15
			100 N	648.0214	50.03079	15
			150 N	925.1943	54.20093	15
	unilateral loading (right side)	not splinted	50 N	920.9272	135.02035	15
			100 N	1204.5317	128.14341	15
			150 N	1360.1664	68.99931	15
		splinted	50 N	709.4391	23.57164	15
			100 N	902.5915	18.17774	15
			150 N	1068.4393	59.32628	15
Left distal edentulous area	Bilateral loading	not splinted	50 N	500.4302	504.67803	15
			100 N	637.0740	60.60470	15
			150 N	1083.6929	136.50435	15
		splinted	50 N	425.6538	55.92623	15
			100 N	645.2451	79.86795	15
			150 N	959.1810	114.64353	15
	unilateral loading (right side)	not splinted	50 N	582.3127	97.80366	15
			100 N	655.1563	93.15064	15
			150 N	756.9038	76.97516	15
		splinted	50 N	134.8874	12.57018	15
			100 N	274.6258	48.74310	15
			150 N	413.7942	19.96682	15
