# Supplementary material for: Mandibular Overdenture Supported by Two or Four Unsplinted or Two Splinted Ti-Zr Mini-Implants: In Vitro Study of Peri-Implant and Edentulous Area Strains
Source: Biomimetics (Basel). 2024 Mar 15;9(3):178. doi: 10.3390/biomimetics9030178 (PMC10968521; doi:10.3390/biomimetics9030178)
Supplement: Supplementary file 1 [file biomimetics-09-00178-s001.zip › Supplementary Table S6a-d, MANOVA, 2MDI posterior area sa post-hoc.pdf]

Supplementary Table S6. a-d. MANOVA analysis with Effect Sizes (η²) for microstrains recorded from straingauges bonded on the right and left posterior edentulous area in the two-MDI models dependent on Splinting Status, Loading Position and Loading Force

Two-MDI models: MANOVA

Strains from SGs in the Posterior Edentulous Area		Type III Sum of Squares	df	Mean Square	F	*P*	Partial Eta Squared
Corrected Model	Right side	14499250.422[a]	11	1318113.675	208.396	<0.01**	0.932
	Left side	11851735.555[b]	11	1077430.505	39.568	<0.01**	0.722
Intercept	Right side	131614787.990	1	131614787.990	20808.524	<0.01**	0.992
	Left side	62462695.773	1	62462695.773	2293.918	<0.01**	0.932
Loading position	Right side	5361351.461	1	5361351.461	847.639	<0.01**	0.835
	Left side	2569000.319	1	2569000.319	94.346	<0.01**	0.360
Splinting status	Right side	886839.992	1	886839.992	140.211	<0.01**	0.455
	Left side	2319426.465	1	2319426.465	85.180	<0.01**	0.336
Loading force	Right side	6982307.392	2	3491153.696	551.957	<0.01**	0.868
	Left side	4740375.134	2	2370187.567	87.044	<0.01**	0.509
Loading position * Splinting status	Right side	737298.053	1	737298.053	116.568	<0.01**	0.410
	Left side	1200372.879	1	1200372.879	44.083	<0.01**	0.208
Loading position * Loading force	Right side	336436.258	2	168218.129	26.596	<0.01**	0.240
	Left side	913271.536	2	456635.768	16.770	<0.01**	0.166
Splinting status * Loading force	Right side	138317.761	2	69158.880	10.934	<0.01**	0.115
	Left side	43133.207	2	21566.603	.792	0.455	0.009
Loading position * Splinting status * Loading force	Right side	56699.505	2	28349.753	4.482	0.013*	0.051
	Left side	66156.016	2	33078.008	1.215	0.299	0.014
Error	Right side	1062607.058	168	6325.042			
	Left side	4574589.532	168	27229.700			
Total	Right side	147176645.470	180				
	Left side	78889020.860	180				
Corrected Total	Right side	15561857.480	179				
	Left side	16426325.087	179				

a. R Squared = .932 (Adjusted R Squared = .927)

b. R Squared = .722 (Adjusted R Squared = .703)

df=degree of freedom; *P*=Level of significance; **<0.01; *=<0.05

Supplementary Table 6 b. The significance of the differences of microstrains in the 2-MDI models recorded from strain gauges on the right and left edentulous area under denture saddles dependent on two different loading positions on the respective overdentures

Two MDI models, Posterior Edentulous area: Loading position

Edentulous area	(I) Loading position	(J) Loading place	Mean Difference (I-J)	Std. Error	P^{b}	95% Confidence Interval for Difference[b]	
						Lower Bound	Upper Bound
Right side	bilateral	unilateral (right side)	-345.168*	11.856	<0.01**	-368.574	-321.763
	unilateral (right side)	bilateral	345.168*	11.856	<0.01**	321.763	368.574
Left side	bilateral	unilateral (right side)	238.933*	24.599	<0.01**	190.370	287.496
	unilateral (right side)	bilateral	-238.933*	24.599	<0.01**	-287.496	-190.370

Based on estimated marginal means

*. The mean difference is significant at the .05 level.

b. Adjustment for multiple comparisons: Sidak.

P=Level of significance; **<0.01; *=<0.05

Supplementary Table 6 c. Significance of the differences between microstrains recorded from strain gauges on the right and left edentulous area under denture saddles in the 2-MDI models dependent on Splinting status

Two MDI models, Posterior Edentulous area: Splinting status

Edentulous area	(I) splintano	(J) splintano	Mean Difference (I-J)	Std. Error	$P.^{b}$	95% Confidence Interval for Difference[b]	
						Lower Bound	Upper Bound
Right side	not splinted	splinted	140.384*	11.856	<0.01**	116.978	163.789
	splinted	not splinted	-140.384*	11.856	<0.01**	-163.789	-116.978
Left side	not splinted	splinted	227.030*	24.599	<0.01**	178.468	275.593
	splinted	not splinted	-227.030*	24.599	<0.01**	-275.593	-178.468

Based on estimated marginal means

*. The mean difference is significant at the .05 level.

b. Adjustment for multiple comparisons: Sidak.

P=Level of significance; **<0.01

Supplementary Table 6 d. Post-hoc tests for the significance of the differences between microstrains in the 2-MDI models recorded from strain gauges on the right and left edentulous area under denture saddles dependent on different Loading forces

Two MDI models, Posterior Edentulous area: post-hoc tests among loading forces

Edentulous area	(I) Force	(J) Force	Mean Difference (I-J)	Std. Error	*P*.[b]	95% Confidence Interval for Difference[b]	
						Lower Bound	Upper Bound
Right side	50 N	100 N	-222.686*	14.520	<0.001**	-257.706	-187.667
		150 N	-481.972*	14.520	<0.001**	-516.991	-446.953
	100 N	50 N	222.686*	14.520	. <0.001**	187.667	257.706
		150 N	-259.286*	14.520	<0.001**	-294.305	-224.266
	150 N	50 N	481.972*	14.520	<0.001**	446.953	516.991
		100 N	259.286*	14.520	<0.001**	224.266	294.305
Left side	50 N	100 N	-142.204*	30.127	<0.001**	-214.865	-69.544
		150 N	-392.572*	30.127	<0.001**	-465.232	-319.912
	100 N	50 N	142.204*	30.127	<0.001**	69.544	214.865
		150 N	-250.368*	30.127	<0.001**	-323.028	-177.707
	150 N	50 N	392.572*	30.127	<0.001**	319.912	465.232
		100 N	250.368*	30.127	<0.001**	177.707	323.028

Based on estimated marginal means

*. The mean difference is significant at the .05 level.

b. Adjustment for multiple comparisons: Sidak.

P=Level of significance; **<0.01
