# Supplementary material for: Mandibular Overdenture Supported by Two or Four Unsplinted or Two Splinted Ti-Zr Mini-Implants: In Vitro Study of Peri-Implant and Edentulous Area Strains
Source: Biomimetics (Basel). 2024 Mar 15;9(3):178. doi: 10.3390/biomimetics9030178 (PMC10968521; doi:10.3390/biomimetics9030178)
Supplement: Supplementary file 1 [file biomimetics-09-00178-s001.zip › Supplementary Table S7a-d distalna 4 mdi with MANOVA and post-hoc.pdf]

Supplementary Table S7a. Four single unit (unsplinted) MDI model; microstrains from the right and left distal edentulous area (under mandibular overdenture saddles) during bilateral and unilateral (right side) loadings with 50 N, 100 N, and 150 N forces applied at the second premolar and first molar area

Straingauge position	Loading Force	Loading place	Mean	Std. Deviation	N
Distal edentulous area: right side	50 N	bilaterally	400.86	55.53	15
		unilaterally (right side)	739.48	70.94	15
	100 N	*bilaterally*	835.99	55.33	15
		unilaterally (right side)	893.93	68.64	15
	150 N	bilaterally	924.15	70.67	15
		unilaterally (right side)	1019.08	59.43	15
Distal edentulous area: left side	50 N	bilaterally	494.38	74.63	15
		unilaterally (right side)	413.15	63.84	15
	100 N	bilaterally	811.99	59.81	15
		unilaterally (right side)	475.09	70.94	15
	150 N	bilaterally	904.81	64.31	15
		unilaterally (right side)	547.00	113.19	15

Supplementary Table S7b. MANOVA with Effect Sizes (η²) for microstrains recorded from straingauges bonded on the right and left posterior edentulous area in the four-MDI model dependent on Splinting Status and Loading Position

Strains registered from posterior edentulous area during overdenture loading in the Four-MDI single-unit model: MANOVA

Source	Posterior edentulous area	Type III Sum of Squares	df	Mean Square	F	Sig.	Partial Eta Squared
Corrected Model	Right side	3547086.343[a]	5	709417.27	174.34	<0.01**	0.912
	Left side	3029551.821[b]	5	605910.36	103.27	<0.01**	0.860
Intercept	Right side	57923804.307	1	57923804.31	14234.45	<0.01**	0.994
	Left side	33240974.984	1	33240974.98	5665.49	<0.01**	0.985
Loading position	Right side	603892.689	1	603892.69	148.40	<0.01**	0.639
	Left side	1505211.083	1	1505211.08	256.54	<0.01**	0.753
Loading force	Right side	2594347.523	2	1297173.76	318.77	<0.01**	0.884
	Left side	1168586.088	2	584293.04	99.59	<0.01**	0.703

Loading position * Loading force	Right side	348846.130	2	174423.07	42.86	<0.01**	0.505
	Left side	355754.650	2	177877.33	30.32	<0.01**	0.419
Error	Right side	341818.558	84	4069.27			
	Left side	492850.574	84	5867.27			
Total	Right side	61812709.208	90				
	Left side	36763377.378	90				
Corrected Total	Right side	3888904.901	89				
	Left side	3522402.395	89				

a. R Squared = .912 (Adjusted R Squared = .907)
df=degree of freedom, *P*=Level of significance; **=p<0.01

Supplementary Table S7c. The significance of the differences between microstrains recorded from the right and left edentulous area under denture saddles in the 4-MDI model when the overdenture was loaded at two different loading positions

Significance of the differences for mean microstrain values registered from posterior edentulous area in the four-MDI unsplinted model dependent on Loading position

Dependent Variable	Loading position		Mean Difference (I-J)	Std. Error	*P*.[b]	95% Confidence Interval for Difference[b]	
						Lower Bound	Upper Bound
Right side	bilateral	unilateral (right side)	-163.828*	13.45	<0.01**	-190.57	-137.08
	unilateral (right side)	bilateral	163.828*	13.45	<0.01**	137.08	190.57
Left side	bilateral	unilateral (right side)	258.647*	16.15	<0.01**	226.53	290.76
	unilateral (right side)	bilateral	-258.647*	16.15	<0.01**	-290.76	-226.53

Based on estimated marginal means
*. The mean difference is significant at the .05 level.
b. Adjustment for multiple comparisons: Sidak.
Df=degree of freedom, *P*=Level of significance; **=p<0.01

Supplementary Table S7d. The significance of the differences (post-hoc tests) between microstrains in the 4-MDI model recorded from strain gauges on the right and left edentulous area under denture saddles when the overdenture was loaded with three different loading forces

Post hoc tests for loading force (strains from posterior edentulous area in the four-MDI unsplinted model)

Dependent Variable	Loading Force		Mean Difference (I-J)	Std. Error	$P.^b$	95% Confidence Interval for Difference[b]	
						Lower Bound	Upper Bound
Right side posterior edentulous area	50 N	100 N	-294.791*	16.47	<0.01**	-334.92	-254.66
		150 N	-401.445*	16.47	<0.01**	-441.57	-361.32
	100 N	50 N	294.791*	16.47	<0.01**	254.66	334.92
		150 N	-106.654*	16.47	<0.01**	-146.78	-66.53
	150 N	50 N	401.445*	16.47	<0.01**	361.32	441.57
		100 N	106.654*	16.47	<0.01**	66.53	146.78
Left side posterior edentulous area	50 N	100 N	-189.776*	19.78	<0.01**	-237.96	-141.59
		150 N	-272.140*	19.78	<0.01**	-320.32	-223.96
	100 N	50 N	189.776*	19.78	<0.01**	141.59	237.96
		150 N	-82.363*	19.78	<0.01**	-130.55	-34.18
	150 N	50 N	272.140*	19.78	<0.01**	223.96	320.32
		100 N	82.363*	19.78	<0.01**	34.18	130.55

Based on estimated marginal means

*. The mean difference is significant at the .05 level.

b. Adjustment for multiple comparisons: Sidak.

df=degree of freedom, *P*=Level of significance; **=p<0.01
